# EspH interacts with the host active Bcr related (ABR) protein to suppress RhoGTPases

**DOI:** 10.1080/19490976.2022.2130657

**Published:** 2022-10-11

**Authors:** Rachana Pattani Ramachandran, Ipsita Nandi, Nir Haritan, Efrat Zlotkin-Rivkin, Yael Keren, Tsafi Danieli, Mario Lebendiker, Naomi Melamed-Book, William Breuer, Dana Reichmann, Benjamin Aroeti

**Affiliations:** a Department of Cell and Developmental Biology, Alexander Silberman Institute of Life Sciences, The Hebrew University of Jerusalem, Jerusalem, Israel; b The Protein Production Facility, Wolfson Centre for Applied Structural Biology, Alexander Silberman Institute of Life Sciences, The Hebrew University of Jerusalem, Jerusalem, Israel; c Bioimaging Unit, Alexander Silberman Institute of Life Sciences, The Hebrew University of Jerusalem, Jerusalem, Israel; d Proteomics and Mass Spectrometry Unit, The Alexander Silberman Institute of Life Sciences, The Hebrew University of Jerusalem, Jerusalem, Israel; e Department of Biological Chemistry, The Alexander Silberman Institute of Life Sciences, The Hebrew University of Jerusalem, Jerusalem, Israel

**Keywords:** Enteropathogenic *E. coli*, Type III secreted effectors, EspH, Rho GTPases, active Bcr related (ABR), host-pathogen interactions

## Abstract

Enteropathogenic *Escherichia coli* are bacterial pathogens that colonize the gut and cause severe diarrhea in humans. Upon intimate attachment to the intestinal epithelium, these pathogens translocate via a type III secretion system virulent proteins, termed effectors, into the host cells. These effectors manipulate diverse host cell organelles and functions for the pathogen’s benefit. However, the precise mechanisms underlying their activities are not fully understood despite intensive research. EspH, a critical effector protein, has been previously reported to disrupt the host cell actin cytoskeleton by suppressing RhoGTPase guanine exchange factors. However, native host proteins targeted by EspH to mediate these activities remained unknown. Here, we identified the active Bcr related (ABR), a protein previously characterized to possess dual Rho guanine nucleotide exchange factor and GTPase activating protein (GAP) domains, as a native EspH interacting partner. These interactions are mediated by the effector protein’s C-terminal 38 amino acid segment. The effector primarily targets the GAP domain of ABR to suppress Rac1 and Cdc42, host cell cytotoxicity, bacterial invasion, and filopodium formation at infection sites. Knockdown of ABR expression abolished the ability of EspH to suppress Rac1, Cdc42. Our studies unravel a novel mechanism by which host RhoGTPases are hijacked by bacterial effectors.

## Introduction

Enteropathogenic *Escherichia coli* (EPEC), one of the most important human diarrheagenic bacterial pathogens, infects people mainly in low and middle-income countries.^1^ In contrast, the closely related enterohemorrhagic *Escherichia coli* (EHEC), which causes hemorrhagic colitis and hemolytic uremic syndrome in humans, is prevalent mainly in the industrial world.^2,3^ *Citrobacter rodentium (C. rodentium)*, a natural mouse pathogen that employs similar strategies of colonization and pathogenesis, serves as an *in vivo* model for studying EPEC and EHEC infection.^4^ Following attachment to the host cell surface, these pathogens utilize the type III secretion system (T3SS) to introduce bacterial proteins, termed ‘effector’ proteins, into the host cells.^5,6^ These effectors specifically target and manipulate host cell organelles and signaling pathways, leading to intimate binding of the bacteria to host enterocytes via the attaching and effacing (A/E) lesion formation,^7^ modulation of host cell death pathways,^4,8^ and inhibition of host immune responses.^9^ Recent *in vivo* studies using the *C. rodentium* model have shown that effectors act as a multifunctional and interconnected network within the host cells. These characteristics are essential for inducing the diarrheal disease.^10,11^

EPEC, EHEC, and *C. rodentium* inhibit their invasion (phagocytosis) into the host cells and are therefore defined as extracellular pathogens. Several effectors have been assigned to mediate this activity (e.g., EspJ, EspF, EspB),^12–18^ one of which is EspH.^13,19–22^ Once translocated, EspH disrupts the actin cytoskeleton; this results in dramatic cell cytotoxicity, cell rounding, and detachment, likely due to disruption of cell adhesion molecules.^19,21,23^ Additional reported EspH-dependent effects include the suppression of actin-rich filopodia formed at infection sites and induction of pedestal elongation,^19,22^ activation of caspase-3,^21^ perturbation of tight junctions,^24^ and inhibition of the mitogen-activated protein (MAP) kinase signaling pathway.^25^

The Rho family of small GTPases (Rho, Rac and Cdc42) regulate a broad range of cellular responses, including alterations in cell adhesion, phagocytosis, cell protrusions and polarity, and more. RhoGTPases modulate these activities by controlling diverse cellular and molecular mechanisms, including signal transduction pathways via MAP kinases, the linking of signaling membrane receptors to the actin cytoskeleton, and the regulation of gene transcription.^26,27^ Hence, it would have been conceivable to postulate that many of the EspH-dependent functions are attributed to the ability of the effector to modulate critical regulators of the actin cytoskeleton, such as the RhoGTPases.^13,21,23^ However, the mechanism by which EspH targets RhoGTPases is not entirely understood. Studies have shown that EspH can inactivate host Rho guanine nucleotide exchange factors (GEFs) by binding their tandem Dbl-homology (DH) and the adjacent pleckstrin-homology (PH) domains.^13^ It has been reasoned that by targeting the DH-PH domains, RhoGTPase inactivation is achieved by the continuous GTPase-activating protein (GAP) activity, causing accelerated hydrolysis of GTP to GDP that eventually switches off the enzyme.^21^ However, the ability of EspH to bind RhoGEFs was demonstrated for exogenously expressed proteins (e.g., p115-RhoGEF), leaving the natural Rho regulators targeted by the effector protein unidentified.^13^

In this study, we discovered the mechanism by which EspH targets RhoGTPases. ABR (active Bcr related), which is structurally similar to Bcr, is a 97.598 kDa protein with a unique structure, harboring two opposing activities that regulate RhoGTPases: a DH-PH RhoGEF domain positioned close to its N-terminus and a RhoGAP domain juxtaposed to the C-terminus. Additionally, ABR possesses a phospholipid-binding C2 domain positioned between the two domains and a PDZ binding motif located at the C-terminus of the protein.^28–31^ The DH-PH domain displays an *in vitro* GEF activity toward the Rho GTPases Cdc42, Rac1, Rac2, and RohA.^30^ The GAP domain of ABR (and Bcr) acts on Rac1/Rac2 and Cdc42, but not RhoA.^30,32,33^ Interestingly, the GAP and GEF domains of ABR bind the GTPases in a noncompetitive manner, suggesting that the two domains may bind simultaneously two RhoGTPases and regulate their function independently.^30^ Notably, the ABR effect on Rac1, Cdc42, and RhoA was also demonstrated *in vivo*.^33–37^ ABR has also been implicated in the negative regulation of phagocytosis, cell dissociation, apoptosis, and inflammation.^33,34,38^ Here we demonstrate for the first time that host ABR is a significant interacting partner of EspH. These interactions are exploited to down-regulate Rac1 and Cdc42 in EPEC-infected cells. Moreover, we show that this activity is exerted by targeting the GAP domain of ABR and that the EspH-ABR interaction facilitates the inhibition of bacterial invasion into the epithelial cells and the appearance of transient filopodia at infection sites.

## Results

### ABR is a major binding partner of EspH

HeLa cells were infected with EPEC-*∆espH*, EPEC-*∆espH*/pEspH*_wt_*, or EPEC-*∆espH*/pEspH_Δ*130-168*_ under conditions permitting efficient effector translocation yet preserving host cell adherence to the tissue culture plate [see Materials and Methods and ref^25^]. Co-precipitation combined with label-free quantitative (LFQ) proteomic analysis identified several host cell proteins that were specifically pulled down with high confidence with EspH*_wt_* compared to cells infected with the EPEC-∆*espH* mutant (Table S1a). Among them, the RhoGTPase regulator ABR was by far the most abundant protein, enriched >1000 fold compared to other proteins that were enriched ~100-fold or less (Table S1a). These results suggest that ABR could be a significant interactor of EspH.

**Figure 1.**
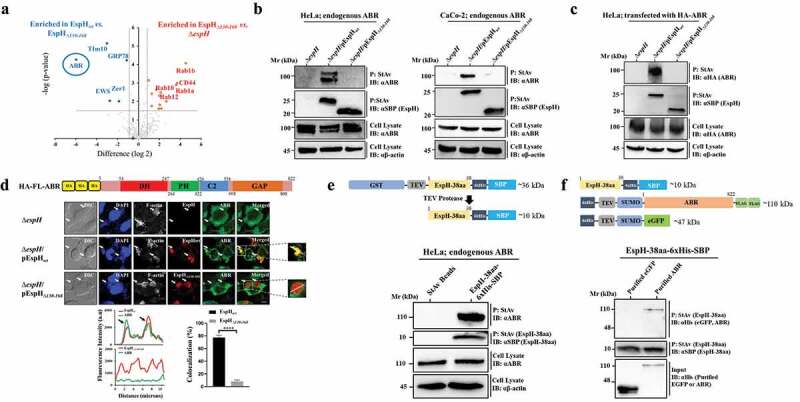
Translocated EspH interacts with host cell ABR, and these interactions depend on the EspH-38aa segment of the effector protein. (a) ***Identification of ABR as a major EspH binding partner.*** HeLa cells were infected with EPEC-∆*espH*, EPEC-∆*espH*/pEspH*_wt_*, or EPEC-*∆espH*/pEspH_∆*130-168*_ for 90 min at 37°C. EspH expression was induced, and cells were subjected to co-precipitation experiments. Interacting proteins were analyzed by liquid chromatography coupled with tandem mass spectrometry (LC-MS/MS). Label-free quantification (LFQ) was applied to compare the coprecipitated proteins upon incubation with bacteria lacking or expressing the EspH variants. The experiment was repeated four times, and the median fold changes are shown in **Tables sS1a-c**. Volcano plots of differentially abundant proteins identified in the EspH*_wt_* vs. EspH_∆*130-168*_ coprecipitates (left blue; see **Table S1c**) and EspH_∆_*_130-168_* vs. Δ*espH* coprecipitates (right red; see **Table S1b**) identified by mass spectrometry, are shown. The differential significance was defined based on FDR < 0.05 and a fold change greater than 2 (indicated with dashed lines). The results identify ABR (encircled) as a major protein that coprecipitates with EspH*_wt_*, but not with EspH_∆*130-168*_. (b) ***Endogenous ABR coprecipitates with translocated EspH_wt_, but not with EspH_∆130-168_, in HeLa and Caco-2 cells***. HeLa (left) and Caco-2_BBe_ (right) cells were infected with the indicated EPEC strains, and pulldown experiments were performed. The SBP-tagged EspH was precipitated (p) with StAv agarose beads, and the precipitated effector was identified by SDS-PAGE followed by immunoblotting (IB) using anti-SBP antibodies. Co-precipitated ABR was detected by probing with anti-ABR antibodies. ABR expression levels and cell lysate protein loading was evaluated by anti-ABR and anti-β-actin antibodies, respectively. A representative immunoblot (of 3 independent experiments) is shown. (c) ***HA-tagged ABR expressed in HeLa cells coprecipitates with translocated EspH_wt_ but not with EspH_∆130-168_.*** HA-tagged ABR was ectopically expressed in HeLa cells. Cells were then infected with the indicated EPEC strains, lysed, and EspH was precipitated using StAv beads. The presence of precipitated EspH (P) and coprecipitated HA-ABR was identified by IB using anti-HA tag antibodies. HA-ABR expression levels and cell lysate protein loading in cell lysates were evaluated by anti-ABR and anti-β-actin antibodies, respectively. A representative gel (of 3 independent experiments) is shown. (d) ***ABR co-clusters with translocated EspH_wt_, but not with EspH_∆130-168_.*** HA-tagged full-length-(FL)-ABR (schematically presented in the **upper panel**) was expressed in HeLa cells, and the cells were infected with the indicated EPEC strains. Cells were then fixed, permeabilized and immunostained with anti-HA (ABR) or anti-SBP (EspH) antibodies. Cells were also stained with DAPI and Texas Red Phalloidin to visualize DNA (host nuclei and bacterial microcolonies) and the F-actin cytoskeleton (pedestals at infection sites), respectively. Differential interference contrast (DIC) images are also shown. Representative confocal images (out of 3 independent experiments) are shown (**middle panel**). Arrows point toward infecting microcolonies. Scale bar = 5 μm. Fluorescence intensity profiles (**lower left panel**) were generated along a drawn line over EspH-ABR labeled regions, as exemplified in the boxed areas of the confocal images. Arrows point to regions of overlapping fluorescence green and red signals, and therefore to sites of protein colocalization. The percentage of colocalization was determined (**lower right panel**). Results are mean ± SE of 20 measurements. **** P < .0001 was analyzed by unpaired two-tailed t test. (e) ***Endogenous ABR coprecipitates with the EspH-38aa fragment.*** A purified EspH38aa-6xHis-SBP fragment (schematically presented in the upper panel. **See also Figure S2**) was used for pulling down ABR from HeLa cell lysates. A representative gel (of 3 independent experiments) is shown. (f) ***Purified ABR coprecipitates with the EspH-38aa fragment.*** A purified EspH38aa-6xHis-SBP fragment was used to pulldown recombinantly expressed and purified 6xHis-TEV-SUMO-ABR-2xFLAG (**Figure S3**) or recombinantly expressed and purified 6xHis-TEV-SUMO-eGFP (**Figure S4)**. A representative gel (of 3 independent experiments) is shown.

We have shown that the C-terminal 38-aa segment of EspH (EspH-38aa segment) is predicted to contain secondary structures and is important for mediating EspH-dependent MAP kinase inhibition.^25^ Interestingly, when a similar co-precipitation combined with the LFQ proteomic analyses was performed using cells infected with EPEC-∆*espH*/pEspH_∆*130-168*_, ABR was not detected among the interactors (Table S1b). Comparative analysis of abundance levels of proteins that coprecipitated with EspH*_wt_* vs. EspH_∆*130-168*_ also identified ABR as a protein that coprecipitated specifically with EspH*_wt_* at the highest strength (Figure 1a, left blue, Table S1c), but not at all with EspH_∆*130-168*_ (Figure 1a right red, Table S1b), suggesting that the presence of the EspH-38aa segment is important for EspH binding to host ABR.

Notably, other host proteins that coprecipitated specifically with EspH also showed binding capacity that is dependent on the presence of the EspH-38aa segment (e.g., Tim10, EWSR1, ZER1, and GRP78). However, these proteins were significantly less abundant than ABR (Figure 1a left blue and Table S1c), further suggesting that ABR is a strong interactor of EspH. Other proteins (e.g., the Rab10, Rab12, Rab3A GTPases) that specifically coprecipitated with EspH*_wt_* (Table S1a) were even more abundant in the EspH_∆*130-168*_ (Figure 1a right red and Table S1B), suggesting that the Rab proteins bind regions upstream to the C-terminal 38-aa segment of EspH and that in the absence of this segment, their binding to the mutant effector is even stronger compared to the WT effector (Table S1c). The STRING protein-protein annotation indicates that although none of the coprecipitated proteins were predicted to be ABR binding partners, the coprecipitated Rab GTPases were predicted to form a highly connected network among themselves (Figure S1).

Co-precipitation followed by IB analyses confirmed the dependence of EspH-ABR interactions on the EspH-38aa segment in HeLa and Caco-2_BBe_ cells (Figure 1b). Notably, the endogenous ABR was co-precipitated as two bands from HeLa cells lysates. This could be attributed to the expression of alternatively spliced isoforms, one that is shorter than the canonical long (~100 kDa) isoform [see: https://www.uniprot.org/uniprot/Q12979#Q12979-1 and ref^39^]. Similar results were obtained with HeLa cells ectopically expressing an HA-tagged of the long (~100 kDa) ABR isoform (Figure 1c). Immunofluorescence analyses showed that the ectopically expressed ABR co-clusters with translocated EspH*_wt_*, but not with EspH_∆*130-168*_ at bacterial infection sites (Figure 1d). These results further suggest that EspH interacts with ABR and that the EspH-38aa segment plays a role in the process.

Finally, we asked whether the EspH-38aa segment can interact with ABR. Initially, pulldown assays using purified glutathione s transferase (GST)-EspH-38aa-6xHis-streptavidin (StAv) binding peptide (SBP) coupled to StAv beads failed to coprecipitate the endogenous ABR from HeLa cells (data not shown). This has raised the possibility that the N-terminally fused GST interfered with ABR binding. Therefore, similar pulldown experiments were performed after GST removal by the tobacco etch virus (TEV) protease cleavage, i.e., using purified EspH-38aa-6xHis-SBP (Figure S2) as a bait. Indeed, unlike the StAv beads alone, EspH-38aa-6xHis-SBP coupled to StAv beads precipitated the ABR from cell lysates (Figure 1e). Similarly, the EspH-38aa-6xHis-SBP fragment could coprecipitate the purified human 6xHis-TEV-SUMO-ABR-2xFLAG (Figure S3), but not the purified 6xHis-TEV-SUMO-eGFP (Figure S4) control (figure 1f). Taken together, these results suggest that translocated EspH interacts firmly with ABR and that the C-terminal 38-aa segment of the effector plays a role in mediating these interactions.

### Ectopically expressed EspH-eGFP interacts and colocalizes with ABR in an EspH-38aa dependent manner

**Figure 2.**
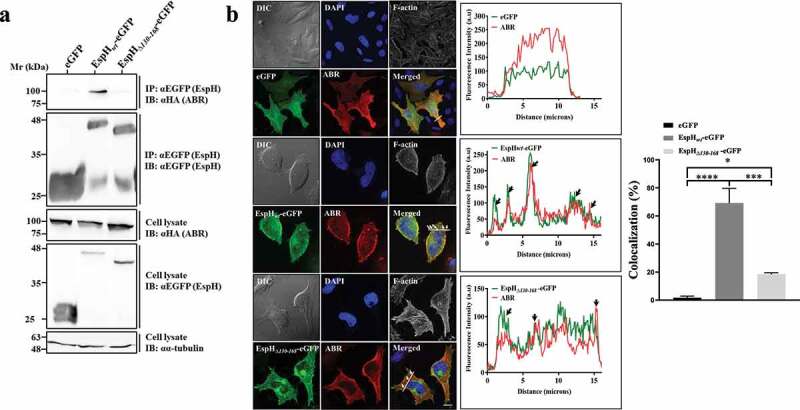
Ectopically expressed EspH interacts with HA-tagged ABR. (a) ***HA-tagged ABR coprecipitates with EspH_wt_-eGFP in an EspH-38aa dependent manner***. HeLa cells were co-transfected with the indicated eGFP and the HA-tagged ABR encoding constructs, lysed, and eGFP was immunoprecipitated (IP). The presence of the IP EspH and the co-IP’ed ABR was detected by IB, using anti-eGFP and anti-HA antibodies, respectively. The protein level in cell lysates was evaluated by IB with anti-α-tubulin antibodies. A representative gel (of 3 independent experiments) is shown. (b) ***EspH_wt_-eGFP and HA-tagged ABR colocalize primarily in peripheral cell sites.*** HeLa cells were co-transfected with the indicated eGFP and HA-ABR encoding constructs. Cells were fixed, permeabilized, and then immunostained with anti-HA (ABR) antibodies. Cells were also stained with DAPI and Phalloidin CF 647 to visualize cell nuclei and F-actin, respectively. DIC images are also shown. Arrows point to regions of colocalization. Representative confocal images (out of 3 independent experiments) are shown **(lef)**. Scale bar = 20 μm. Fluorescence intensity profiles were generated **(middle)** along a drawn line over EspH-ABR co-labeled areas, as exemplified in the confocal images. The percent of colocalization was determined as in Figure 1 **(righ)**. Results are mean ± SE of 20 measurements * P < .05, *** P < .001, **** P < .0001 was analyzed by one-way ANOVA with Bonferroni’s correction.

We asked whether ectopically expressed EspH can interact with ABR. The existence of such an interaction would imply that EspH can target ABR autonomously, i.e., independent of any bacterial components. To address this hypothesis, eGFP, or EspH*_wt_*-eGFP, or EspH_Δ*130-168*_-eGFP encoding constructs were co-transfected with HA-tagged ABR in HeLa cells, and the interaction between the expressed proteins was analyzed by co-immunoprecipitation (co-IP) followed by IB analysis. The results showed that ABR was co-IPed with EspH*_wt_*-eGFP but not with eGFP alone or EspH_Δ*130-168*_-eGFP (Figure 2a). Confocal imaging showed that expression of EspH*_wt_*-eGFP partially colocalized with ABR. Conversely, the colocalization of eGFP, or EspH_Δ*130-168*_-eGFP, with ABR was minimal (Figure 2b). These results suggest that EspH can interact with ABR autonomously and that its C-terminal 38-aa segment is essential for mediating these interactions.

### *Translocated EspH_wt_*, *but not EspH*_Δ__*130-168*_, *confers Rac1/Cdc42 inhibition*

**Figure 3.**
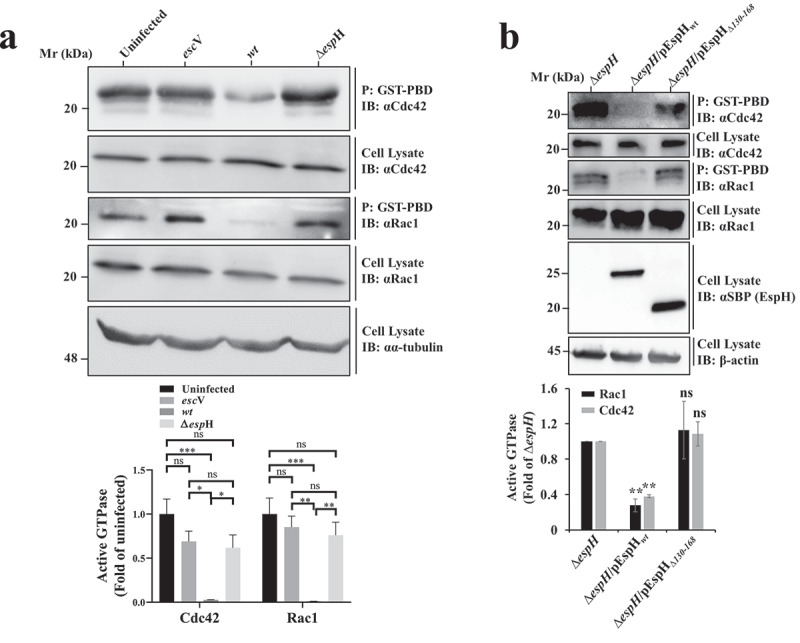
EspH inhibits Rac1 and Cdc42 activity in an EspH-38aa-dependent manner. (a) ***Rac1 and Cdc42 activity levels of EPEC infected cells were determined by a pulldown assay*.** HeLa cells were infected with the indicated EPEC strains. Active Rac1/Cdc42 levels were determined by the GST pulldown-based assay. Active Rac1/Cdc42 precipitated (p) from the cell lysates with the GST-PBD beads was detected by IB with anti-Rac1 or anti-Cdc42 antibodies, respectively. Cell lysates were immunoblotted with the same anti-Rac1 or anti-Cdc42 antibodies, and anti-α-tubulin antibodies were used for estimating lysate protein levels. A representative gel (out of 3 independent experiments) is shown (**upper**). The level of active GTPases was normalized to the level Rac1/Cdc42 in the cell lysate (**lower**). The values obtained for the EPEC-*escV, wt* or ∆*espH* infected cells were further normalized to the level obtained for the uninfected cells (**lower)**. Results are mean ± SE of 3 independent experiments. * P < .05, ** P < .01, *** P < .001, ns, non-significant P ≥ .05 was analyzed by two-way ANOVA with Bonferroni’s correction. (b) ***EspH-38aa dependent inhibition of Rac1 and Cdc42 activity.*** HeLa cells were infected with the indicated EPEC strains. Active Rac1/Cdc42 levels were determined by the GST-pulldown-based assay as explained in panel A. EspH presence in cell lysates was detected with anti-SBP antibodies. A representative gel (out of 3 independent experiments) is shown in the **upper panel**. The level of active GTPases normalized to the level Rac1/Cdc42 in the cell lysates is shown in the **lower panel**. The values obtained for the EPEC-∆*espH*/pEspH*_wt_* or EPEC-∆*espH*/pEspH_∆*130-168*_ infected cells were further normalized to the level obtained for the EPEC-∆*espH* infected cells (**lower)**. Results are mean ± SE of 3 independent experiments. ** P < .01, ns, non-significant P ≥ .05 was analyzed by two-way ANOVA with Bonferroni’s correction.

Using GST-pulldown based assays we could demonstrate that infection with EPEC-*wt*, but not with EPEC-*escV* (*escV*::Tn5kan; T3SS deficient), or EPEC-∆*espH*, decreased the Cdc42 and Rac1 activity levels in the infected cells (Figure 3a), suggesting that EspH is the effector that suppresses the RhoGTPases. The observation that EspH interacts with ABR prompted the hypothesis that the effector protein modulates the activity of RhoGTPase by targeting ABR. To initially verify this hypothesis, we examined if translocated EspH can affect the host Cdc42 and Rac1 activity in an EspH-38aa dependent manner. HeLa cells were infected with EPEC-∆*espH*, EPEC-∆*espH*/pEspH*_wt_*, or EPEC-∆*espH*/pEspH_Δ*130-168,*_ and the activity of Cdc42 and Rac1 was monitored by a pulldown-based assay. The results showed decreased Rac1 and Cdc42 levels only in cells infected with EPEC-∆*espH*/pEspH*_wt_* (Figure 3b), confirming that translocated EspH indeed suppresses RhoGTPase activity and that EspH-ABR interactions mediated by EsH-38aa are required for mediating the effect.

### *EspH*_wt_ *interacts with the ABR GAP domain to inhibit Rac1 and Cdc42*

**Figure 4.**
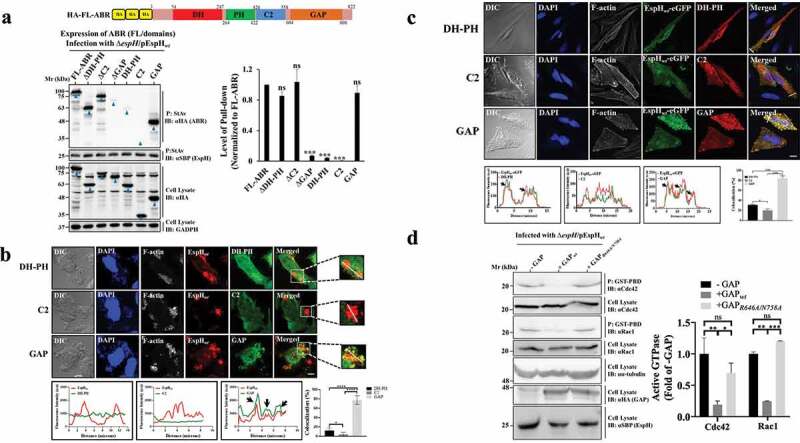
EspH interacts with the GAP domain of ABR to confer RhoGTPase inhibition. (a) ***The HA-GAP of ABR coprecipitates with translocated EspH_wt_***. HA-tagged FL-ABR, ΔDH-PH, ΔC2, ΔGAP, and each of its ABR domains (i.e., DH-PH, C2, and GAP domains, schematically presented in the **upper panel**), were expressed in HeLa cells, which were subsequently infected with EPEC-∆*espH*/pEspH*_wt_*. Cells were lysed and translocated EspH*_wt_* was precipitated (p) with StAv beads. Beads were then subjected to SDS-PAGE followed by IB. Co-precipitated ABR (FL/domains) and the precipitated EspH were detected with anti-HA and anti-SBP antibodies, respectively. Cell lysates were probed with anti-HA antibodies to detect the expressed FL-ABR and its domains and with anti-GAPDH to evaluate the lysate protein loading. A representative gel (out of 3 independent experiments) is shown (**lower left**). Arrowheads point toward the precipitated and coprecipitated protein bands. Protein band intensities were measured to determine the level of pulled-down (co-precipitated) proteins. Values obtained were normalized to the band intensities of corresponding proteins in cell lysates. The values obtained for each ABR domain were further normalized to FL-ABR (**lower right**). Results are mean ± SE of 3 independent experiments. *** P < .001, ns, non-significant P ≥ .05 was analyzed by one-way ANOVA with Bonferroni’s correction. (b) ***Translocated EspH_wt_ colocalizes with HA-tagged GAP.*** HeLa cells were transfected with HA-tagged DH-PH, C2, or GAP encoding plasmids, and subsequently infected with EPEC-∆*espH*/pEspH*_wt_*. Cells were then fixed, permeabilized, and the ABR domains and the translocated EspH*_wt_* were immunolabeled with anti-HA and anti-SBP antibodies, respectively. Cells were also stained with DAPI and Texas Red Phalloidin to visualize cell nuclei and F-actin, respectively. DIC images are also shown. Representative images of 3 independent experiments are shown (**upper**). Scale bar = 5 µm. Fluorescence intensity profiles were generated along a drawn line over EspH-ABR domains labeled regions, as exemplified in the boxed areas of the confocal images, and the percentage of colocalization was determined (**lower**). Arrows point to regions of colocalization. Results are mean ± SE of 20 measurements. * P < .05, **** P < .0001 was analyzed by one-way ANOVA with Bonferroni’s correction. (c) ***Ectopic EspH_wt_-eGFP colocalizes primarily with HA-GAP of* *ABR***. HeLa cells were co-transfected with HA-tagged DH-PH, or C2, or GAP encoding plasmids along with an EspH*_wt_*-eGFP encoding plasmid for 24 hrs. Cells were then fixed, and the HA tag was immunofluorescently labeled with rabbit anti-HA mAb followed by AlexaFluor594 goat anti-rabbit antibodies. Cells were also stained with DAPI and Phalloidin CF 647 to visualize DNA and F-actin, respectively, and then imaged by confocal microscopy. DIC images are also shown. Representative images (out of 3 independent experiments) are shown (**upper**). Scale bar = 20 µm. Fluorescence intensity profiles were generated along with a drawn line over EspH-ABR domain co-labeled areas, as exemplified in the confocal images, and the percent of colocalization was determined (**lower**). Arrows point toward regions of colocalization. Results are mean ± SE of 20 measurements. * P < .05, *** P < .001 was analyzed by one-way ANOVA with Bonferroni’s correction. (d) ***Translocated EspH_wt_ inhibits Rac1 and Cdc42 in a GAP_wt_-dependent fashion.*** HeLa cells were transfected with HA-tagged GAP*_wt_* (+GAP*_wt_*) or GAP*_R646A/N758A_* (+GAP*_R646A/N758A_*) encoding plasmids. Untransfected cells (-GAP) served as control. Cells were then infected with EPEC-∆*espH*/pEspH*_wt_* and subjected to the RhoGTPase activity assay. Rac1 and Cdc42 were pulled down with the GST-PBD beads and analyzed by IB probed with the respective antibodies. Cell lysates were analyzed by IB probed with the same antibodies and with anti-HA and anti-SBP antibodies for detecting GAP and EspH*_wt_*, respectively. The same immunoblots were also probed with anti-α-tubulin antibodies to evaluate the cell lysate protein loading. A representative gel (out of 3 independent experiments) is shown (**left**). Protein band intensities were measured, and the level of active GTPases was quantified and normalized to the level Rac1/Cdc42 in the cell lysate. The values obtained for GAP*_wt_*, or GAP*_R646A/N758A_*, were further normalized to untransfected (-GAP) cells (**right**). Results are mean ± SE of 3 independent experiments. * P < .05, ** P < .01, *** P < .001, ns, non-significant P ≥ .05 was analyzed by two-way ANOVA with Bonferroni’s correction.

Our next objective was to identify specific domains in ABR (i.e., DH-PH, C2, GAP) targeted by EspH. HeLa cells were transfected with constructs encoding the HA-tagged full-length (FL)-ABR, DH-PH, C2, or GAP domains, and the ABR mutants bearing the respective deletion of each domain (i.e., ΔDH-PH; ΔC2; ΔGAP). Cells were then infected with EPEC-∆*espH*/pEspH*_wt_*, and the effector protein was pulled down from the cells using StAv beads. The coprecipitated ABR was identified by IB using anti-HA antibodies. As expected, FL-ABR coprecipitated efficiently with the translocated effector. Compared to the DH-PH and C2 domain, the GAP domain of ABR coprecipitated far more efficiently with the translocated EspH. Accordingly, high levels of the expressed ΔDH-PH (containing C2 and GAP) and ΔC2 (containing DH-PH and GAP) coprecipitated with the injected effector more efficiently than with ΔGAP (Figure 4a). Confocal imaging showed that only the ectopically expressed GAP domain colocalized extensively with translocated EspH*_wt_* at bacterial infection sites (Figure 4b) and with ectopically expressed EspH*_wt_*-eGFP (Figure 4c). These results suggest that translocated EspH targets primarily the GAP domain of ABR.

We then tested if these interactions are functional, i.e., capable of enforcing Rac1 and Cdc42 inhibition. HeLa cells were either mock-transfected or transfected with a GAP*_wt_*, or GAP*_R646A/N758A_* encoding plasmids. Subsequently, cells were infected with EPEC-∆*espH*/pEspH*_wt_* and subjected to the RhoGTPase activity assay, as before. The results showed significant inhibition of both Rac1 and Cdc42 only in the GAP-expressing cells (Figure 4d). The increased effect compared to cells that do not express the GAP (-GAP) could be the result of increased functional interactions exerted between the translocated EspH*_wt_* and the overexpressed GAP domain. The inhibition was not observed in the GAP*_R646A/N758A_* expressing cells, a mutation previously shown to abolish GAP activity.^33^ Taken together, these data suggest that EspH exerts GAP activity toward Rac1 and Cdc42 by targeting the GAP domain of ABR.

### EspH-ABR interactions inhibit bacterial invasion and filopodium formation

**Figure 5.**
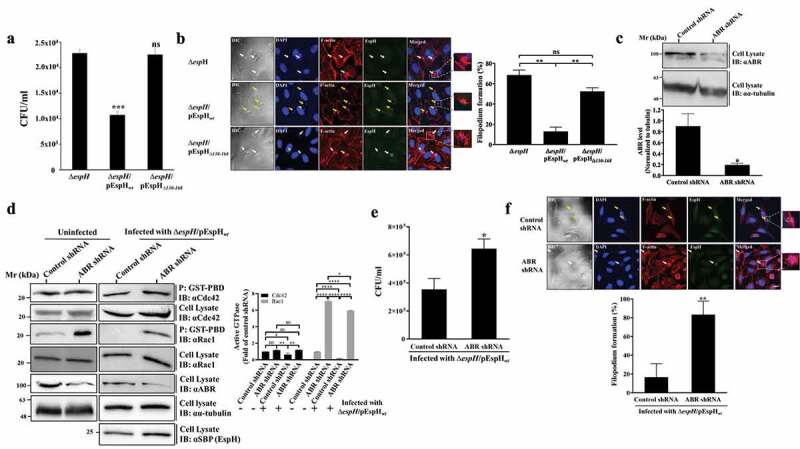
EspH-ABR interactions modulate bacterial invasion and filopodium formation. (a) ***Effects of translocated EspH_wt_ on bacterial invasion***. HeLa cells were infected with the indicated EPEC strains for 90 min at 37°C. The bacterial ‘invasion assay’ was applied to evaluate the degree of bacterial invasion into the cells, as described in Materials and Methods. Results are mean ± SE of 3 independent experiments. *** P < .001, ns, non-significant P ≥ .05 was analyzed by one-way ANOVA with Bonferroni’s correction. (b) ***Translocated EspH*_wt_,*but not EspH*_Δ*130-168*_,*inhibits filopodium formation.*** HeLa cells were infected with the indicated EPEC strains for 15 min at 37°C. The cells were fixed, permeabilized, stained with DAPI (to visualize host nuclei and adhered bacterial microcolonies), Texas Red Phalloidin (to visualize the F-actin cytoskeleton) and anti-SBP (to visualize translocated EspH) and processed for confocal imaging, as described in Materials and Methods. Representative images are shown in the **left panel**. Arrows point toward adherent bacterial microcolonies. White arrows indicate infection sites showing elongated F-actin rich extensions (i.e., filopodia) and yellow arrows indicate point toward infection areas showing F-actin rich foci lacking such extensions (i.e., lacking filopodia). Bar = 5 µm. The percentage of the filopodium formation was calculated by scoring randomly 100 microcolonies (**right panel**). Results are mean ± SE of 3 independent experiments. ** P < .01, ns, non-significant P≥ .05 was analyzed by one-way ANOVA with Bonferroni’s correction. (c) ***Silencing of ABR protein expression.*** HeLa cells expressing stably either Control shRNA or ABR shRNA were isolated as described in Materials and Methods. Cells were lysed and subjected to SDS-PAGE followed by IB analysis using anti-ABR antibodies (for detecting ABR) and anti-α-tubulin antibodies (for evaluating loaded lysate protein levels). A representative immunoblot is shown in the **upper panel** and the ABR expression level normalized to tubulin is shown in the **lower panel**. Results are mean ± SE of 3 independent experiments. * P < .05 was analyzed by unpaired two-tailed t test. (d) ***Effects of ABR expression on Rac1 and Cdc42 activity.*** HeLa cells expressing either Control, or ABR shRNA were analyzed for active Rac1 and Cdc42 levels in uninfected cells (-), and in cells infected with EPEC-Δ*espH*/pEspH*_wt_* for 90 min at 37°C (+) by the GST-pulldown-based assay. Active Rac1/Cdc42 precipitated (p) from the cell lysates with the GST-PBD beads was detected by IB with anti-Rac1 or anti-Cdc42 antibodies, respectively. Cell lysates were immunoblotted with the same anti-Rac1 or anti-Cdc42 antibodies. Anti-α-tubulin antibodies were used for estimating lysate protein levels and probing the lysates with anti-SBP was used for evaluating the host associated EspH levels. Representative gels (out of 3 independent experiments) are shown (**left**). The level of active GTPases was quantified and normalized to the level Rac1/Cdc42 in the cell lysate. The values obtained for ABR shRNA (-), control shRNA (+) or ABR shRNA (+) were further normalized to control shRNA (-) cells (**right**). Results are mean ± SE of 3 independent experiments. * P < .05, ** P < .01, **** P < .0001, ns, non-significant P ≥ .05 was analyzed by two-way ANOVA with Bonferroni’s correction. (e) ***Effects of ABR expression on bacterial invasion***. HeLa cells expressing either Control or ABR shRNA were infected with EPEC-∆*espH*/pEspH*_wt_* for 90 min at 37°C, and an ‘invasion assay’ was performed to evaluate the degree of bacterial invasion into these cells. Results are mean ± SE of 3 independent experiments. * P < .05 was analyzed by unpaired two-tailed t test. (f) ***Effects of ABR expression on filopodia formation.*** HeLa cells expressing either Control or ABR shRNA were infected with EPEC-∆*espH*/pEspH*_wt_* for 15 mins at 37°C. Cells were fixed and stained as in panel B. Representative images from 3 independent experiments are shown (**panel**). Arrows point toward adherent bacterial microcolonies. White arrows indicate infection sites showing elongated F-actin rich extensions reminiscent of filopodia and yellow arrows indicate infection areas showing F-actin rich foci lacking such extensions, i.e., lacking filopodia. Bar = 5 µm. The percentage of the filopodia formation was calculated by scoring 100 microcolonies chosen randomly (**lower**). Results are mean ± SE of 3 independent experiments. ** P < .01 was analyzed by unpaired two-tailed t test.

Studies suggested that EspH blocks Rho activation to antagonize bacterial phagocytosis (invasion) by macrophages.^13^ ABR has also been implicated in negatively regulating phagocytosis.^33^ Studies have also shown that while at an early infection time, EPEC-*wt* or EPEC-∆*espH* induce filopodium formation, EPEC-∆*espH*/pEspH*_wt_* strongly suppresses the filopodium formation.^19,22^ Given these observations, we asked whether the EspH-ABR interactions, shown to downregulate Rac1 and Cdc42, play a role in bacterial invasion and filopodium formation. To investigate the dependence of bacterial invasion on EspH, the ‘invasion assay’ was applied on HeLa cells infected with EPEC-*escV*, EPEC-*wt*, or EPEC-∆*espH*. Infection with EPEC-*wt* resulted in bacterial invasion that is higher than EPEC-*escV* (Figure S5). Invasion levels of EPEC-∆*espH* were even higher than those displayed by EPEC-*wt* (Figure S5), suggesting that translocated EspH indeed suppresses bacterial invasion. To explore the role of EspH-ABR interactions, HeLa cells were infected with EPEC-∆*espH*, EPEC-∆*espH*/pEspH*_wt_*, or EPEC-∆*espH*/pEspH_∆*130-168,*_ and invasion assay was applied, as before. While the invasion levels of EPEC-∆*espH* and EPEC-∆*espH*/pEspH_∆*130-168*_ were comparable, the degree of EPEC-∆*espH*/pEspH*_wt_* invasion was significantly lower (Figure 5a). These results suggest that the inhibitory effect that EspH has on bacterial invasion into HeLa cells depends on its ability to interact with ABR.

To explore the role of EspH-ABR interactions on transient filopodium formation, filopodium formation at infection sites was visualized and scored, as described in Materials and Methods and ref.^19^ While infection with EPEC-∆*espH*/pEspH*_wt_* resulted in a significant reduction in filopodium formation, infection with EPEC-∆*espH*/pEspH_∆*130-168*_ induced filopodium formation levels that are nearly comparable to those imposed by EPEC-∆*espH* (Figure 5b). These results suggest that filopodium formation is also affected by the EspH-ABR interactions.

To further investigate the EspH dependence on ABR, a lentiviral shRNA knockdown approach was used to reduce ABR by ~80% in ABR compared to Control shRNA-treated cells (Figure 5c). Intriguingly, in uninfected cells, the Rac1, but not Cdc42 activity levels were increased in the ABR knockdown compared to the Control shRNA-treated cells (Figure 5d). These results agree with previous studies suggesting that the steady-state levels of active Rac are increased in ABR (and Bcr) deficient cells.^40^ Following infection with EPEC-∆*espH*/pEspH*_wt_*, active Rac1 and Cdc42 levels in the ABR shRNA treated cells were still upregulated, albeit at somewhat reduced levels compared to the uninfected cells (Figure 5d). This could be attributed to residual (~20%) RhoGTPase expression levels left in the ABR shRNA treated cells. Nonetheless, these data argue that ABR is needed for EspH-mediated downregulation of the RhoGTPases active levels.

Next, the EPEC-∆*espH*/pEspH*_wt_* invasion levels into the ABR silenced cells were compared to Control-shRNA cells. The results showed increased invasion levels of EPEC-∆*espH*/pEspH*_wt_* in the ABR shRNA-treated cells (Figure 5e). Filopodium formation was also markedly stimulated on the ABR shRNA-treated cells compared to Control shRNA-treated cells (figure 5f). Altogether, these findings signify the importance of EspH-ABR interactions in modulating Cdc42/Rac1 activity, bacterial invasion, and filopodium formation.

## Discussion

Since its discovery,^19^ numerous studies have shown that EspH displays a strong capacity to modulate the host cell actin cytoskeleton through overriding RhoGTPases.^13,19,21–23^ However, the only attempt to make a mechanistic link between EspH and RhoGTPases was introduced by the Shao team, which suggested that EspH inactivates mammalian RhoGEFs by binding their DH-PH domains.^13^ Yet, the ability of EspH to bind RhoGEFs was demonstrated only for exogenously expressed proteins (e.g., the p115-RhoGEF),^13^ leaving the natural host binding partners of EspH unknown.

Here, we report that ABR, previously characterized to possess dual RhoGEF and RhoGAP regulatory functions,^28–31^ is the natural host target of EspH. Using co-precipitation experiments, we demonstrated that ABR interacts specifically with EspH and that this interaction depends on the C-terminal 38-aa segment of the effector (Figure 1). It is perhaps not surprising that this segment binds the ABR since it has been suggested to contain a highly conserved predicted α-helix,^25^ and helices are well-known to have the potential of mediating protein-protein interactions.^41^ Indeed, the observation that endogenous and purified ABR could coprecipitate with purified EspH-38aa-6xHis-SBP suggests that this effector segment can interact with FL-ABR (Figure 1e). Notably, extensive attempts to produce the FL-EspH in *E. coli* have so far failed, because in all cases, the recombinant protein remained in the insoluble fraction. Therefore, it is currently unknown whether the C-terminal 38aa segment binds ABR in the context of the FL protein. In any case, the capability of EspH to interact with ABR in a C-terminal 38-aa segment dependent fashion seems to be independent of the presence of any bacterial effector (or factor) because the host cell endogenous ABR could be coprecipitated with ectopically expressed EspH*_wt_*-eGFP, but not with EspH_∆*130-168*_-eGFP (Figure 2). Importantly, we show that these interactions are functional because translocated EspH*_wt_*, but not EspH_∆*130-168*_, resulted in Rac1 and Cdc42 inhibition (Figure 3).

ABR includes DH-PH, C2, and GAP domains. In an attempt to gain a deeper insight into the mechanism by which EspH targets the ABR, we asked which of the ABR domains is targeted by the effector protein? To address this question, individual ABR domains were ectopically expressed in HeLa cells, and co-precipitation experiments were carried out after cell infection with EPEC-∆*espH*/pEspH*_wt_*. Results in Figure 4a unambiguously show that the GAP, but not the DH-PH or C2 domains, effectively coprecipitated with the translocated EspH. These results, which were somewhat surprising in light of reports suggesting that EspH targets the DH-PH domain of RhoGEFs,^13^ were further corroborated by data suggesting that translocated EspH*_wt_* reduced the activity of Rac1 and Cdc42 only in GAP*_wt_* but not in the GAP*_R646A/N758A_* deficient mutant-expressing cells (Figure 4d).

Minimal GAP domains typically have low activity levels on their own. Therefore, to gain a function, they have to be stimulated. Indeed, GAPs are regulated by a multi-layered process involving protein-protein and protein-lipid interactions, the binding of second messengers, and/or posttranslational modifications.^42^ The upregulation of the ABR RhoGAP activity may require similar mechanistic elements. Our results suggest that EspH is a predominant factor that stimulates the GAP domain of ABR. As EspH localizes close to the host cell plasma membrane,^19^ the effector may bring the GAP domain of ABR to a closer apposition to the host cell plasma membrane that is enriched with the relevant lipids for its activation. Indeed, the close interplay between RhoGTPases, phosphoinositide metabolism, and the actin cytoskeleton suggests that phosphoinositides at EPEC infection sites^43,44^ may play a role in the EspH-dependent GAP activation.

Accelerating the GTPase activity of Cdc42 and Rac1 through EspH-ABR-GAP interactions may impact multiple processes in the microbe-host interface. For example, EPEC is well known to exert anti-phagocytic activity on professional phagocytes and epithelial cells.^12,14–17,45^ Several effector proteins were suggested to mediate this antagonistic effect,^12^ including EspH.^13^ Here we add a critical mechanistic feature to this EspH activity, which involves its interaction with host ABR (Figure 5). EPEC and EHEC encode several effectors, among them EspT, Map, EspM, and EspG, which have RhoGEF activity.^46–53^ EspH may turn this activity off upon translocation by targeting the host ABR GAP domain. This counterbalancing effect may impact the host cell in many ways; one of them is to limit the microbe localization to the host plasma membrane by antagonizing bacterial invasion.

RhoGTPases have been linked to adaptive and innate immunity processes.^54^ For example, MyD88-independent activation of an actin-Cdc42/Rac pathway is required for Toll-like receptor-stimulated phagocytosis.^55^ Bacterial pathogens that activate Rac1/Cdc42 also activate Rac1/Cdc42-dependent NOD1 signaling and are thereby sensed by the host’s innate immune system.^56^ Importantly, *in vivo* studies have shown that ABR (and Bcr) regulate the innate immune system.^33,34^ Hence, it is possible that in the case of EPEC, inhibition of Rac1/Cdc42 via EspH targeting host ABR serves a dual function: inhibition of bacteria phagocytosis, which allows bacterial escape from host lysosomes and inhibition of innate immunity launched by the host. These combined activities could facilitate successful bacterial colonization and survival on the surface of the infected gut.

Finally, ABR shows detectable expression in many tissues and cell types,^28^ including the human gastrointestinal tract (The Human Protein Atlas; https://www.proteinatlas.org/ENSG00000159842-ABR/tissue). To the best of our knowledge, ABR has not yet been studied in the context of bacterial pathogenesis. Therefore, novel discoveries regarding the mechanism by which bacterial pathogens may target ABR will teach us new and fundamental lessons in the basic cell biology underlying host-pathogen interactions. Furthermore, it may also contribute to the development of new therapeutics to combat bacterial pathogens causing severe human gut illnesses.

## Materials and methods

### Bacterial strains, antibodies, plasmids, primers, and recombinant protein construction

Bacterial strains, antibodies, plasmids, and oligonucleotide primers used in this study are listed in Tables S2-5. The EPEC strains *wt, escV,* ∆*espH,*∆*espH*/pEspH*_wt,_* and *∆espH*/pEspH_Δ*130-168*_ have been previously described.^25^ The EspH-38aa was cloned into GST parallel 1 vector using linearization primers (F&R pGST1 linear and F&R GA 38AA-GST; see Table S5) using the Gibson assembly master mix [NEB # E2611^57^] to obtain the GST-EspH-38aa-6xHis-SBP plasmid (Table S4). The bacterial expression plasmid of FL-ABR (6xHis-TEV-SUMO-ABR-2xFlag; Table S4) was constructed by the Gibson assembly method with the help of F&R Linear sumo primers, F&R GA ABR primers, and gblock flag (Table S5) replacing the eGFP gene segment in pETM11 Sumo3 eGFP vector. The EspH*_wt_*-eGFP and EspH_∆*130-168*_-eGFP encoding plasmids have been described previously (Table S4 and ref^25^). Importantly, the ectopically expressed EspH*_wt_*-eGFP is functional, as it induced host cytotoxicity compared to the eGFP control. Expression of EspH_Δ*130-168*_-eGFP showed intermediate cytotoxicity, i.e. higher than eGFP but lower than EspH*_wt_*-eGFP (Figure S6), a result consistent with the effects observed by translocated EspH*_wt_* (Figure S7). Additional details are presented in the Supplementary Methods. The mammalian expression plasmids bearing HA-tagged ABR domains were constructed using 5’-phosphorylated primers (e.g., HA-ABR_ΔDH-PH_ was created using 16 F’ and 17 R’ primers; Table S5). Mutations were introduced using platinum superfi II DNA polymerase according to the manufacturer’s instructions (https://assets.thermofisher.com/TFS-Assets/LSG/manuals/MAN0014883_Platinum_SuperFi_PCR_MM_UG.pdf, ThermoFisher). The FL-ABR plasmid (Table S4) was used as the template for constructing ABR domain encoding plasmids. All mutations were confirmed by PCR and sequencing.

### Expression and purification of recombinant proteins

All procedures are described in the Supplementary Methods.

### Cell culture

HeLa and CaCo-2_BBe_ cells were cultured as previously described.^25^

### Cell transfections

Typically, plasmid DNA was transiently transfected into HeLa cells (grown to ~70% confluence) for 48 hrs at 37°C, using the TransIT-X2 Transfection Reagent (MIR 6004; Mirus, Madison, WI). In several experiments, HeLa cells cultured on 15 cm plates were transfected with plasmids, using Polyethylenimine Linear (PEI; 1 mg/ml, MW 25000 Polyscience #23966), as follows. A transfection solution was prepared by mixing plasmid DNA (12 µg) with PEI (72 µg) (DNA: PEI 1:6 w/w ratio), followed by incubation for 10 min at 22°C. Cells were washed 2X with PBS. The buffer was then aspirated, leaving the cell monolayer as dry as possible. The transfection solution was added drop-wise to a 70% confluent cell culture while gently swirling the plate. After 3 hrs of incubation in a CO_2_ incubator (37°C; 5% CO_2_; 95% humidity), the transfection solution was replaced with complete DMEM supplemented with 10% fetal calf serum (FCS), 1% glutamine, and 1% penicillin-streptomycin solution and cells were placed in a CO_2_ incubator until use.

### Bacterial pre-activation and infection

Bacterial growth, T3SS pre-activation, EspH expression induced by isopropyl β-D-1 thiogalactopyranoside (IPTG; Promega V395D) and bacterial infection were performed at a multiplicity of infection (MOI) of ~ 100, as previously described.^25^

### ABR silencing by shRNA

Cloning of ABR shRNA into the linearized pLKO.1 puro vector, digestion with AgeI (NEB #R3552) and EcoRI (NEB #R0101S), and ligation of oligonucleotides 27 F’ and 28 R’ into the digested plasmid were performed as described in https://www.addgene.org/protocols/plko/. The nucleotide sequence of the construct was verified. Lentiviruses were produced using the Addgene protocol (https://www.addgene.org/protocols/lentivirus-production/. Briefly, HEK293T cells were seeded (3.8X10^6^ cells/plate) on a 10-cm plate and grown for 24 hrs in a CO_2_ incubator. Then, the cells were treated with 10 µl of 25 µM chloroquine and incubated in CO_2_ incubator for 5 hrs. After 5 hrs, the cells were transfected with mixture of psPAX2 (10 µg), pMD2.G (6 µg) and either pLKO.1-Puro-ABR shRNA (10 µg) or pLKO.1-puro-Scramble (10 µg) plasmids, using PEI (DNA: PEI 1:3 w/w ratio) as the transfection reagent. Viruses were harvested at 24 hrs, 48 hrs and 96 hrs time points, filter sterilized by a 0.45 µm syringe filter and stored at −80°C. For infecting cells, HeLa cells were seeded in a 6-well plate (3x10^5^ cells/well) and incubated for 24 hrs in the CO_2_ incubator. After 24 hrs, cells were infected with 1ml of viral particles harvested after the 96 hrs time point diluted in 1 ml DMEM complete media supplemented with 15 µl protamine sulfate (Sigma #P3369, 10 µg/ml) and incubated for 24 hrs in the CO_2_ incubator. The infection treatment was repeated for three successive days. After the third infection, cells were washed with PBS and selected with complete DMEM media containing 3 ug/ml puromycin for 48 hrs. ABR expression was evaluated by IB.

## Analysis of EspH-ABR interactions by pulldown experiments

### Pulldown experiments analyzed by mass spectrometry

Coprecipitation of host cell proteins with translocated EspH was essentially performed as described for the EspF effector.^58^ HeLa cells, grown to 70% confluency in a Nunclon Delta Treated Square BioAssay Dish (ThermoFisher Scientific #166508), were washed twice with warm (37°C) DMEM and then infected for 90 min at 37°C with pre-activated (DMEM, 3 hrs, 37°C) bacteria and effector protein expression was induced with 0.05 mM IPTG (added to the DMEM during the last 30 min of bacterial activation). Cells were then washed three times with ice-cold PBS and lysed in ice-cold lysis buffer [50 mM Tris (pH-7.4), 150 mM NaCl, 0.5% NP-40] supplemented with protease and phosphatase inhibitors (Sigma, #PPC1010-1 ML). Following 30 min incubation at 4°C, lysates were centrifuged (5,000 g, 15 min, 4°C), and the protein concentration of the supernatants was determined by the BCA protein assay kit (Thermo Scientific #23227). An equal amount (~5 mg) of cell lysate was incubated with 60 µl of StAv Agarose beads (Sigma, #S1638, 50% slurry pre-washed with lysis buffer) for 4 hrs at 4°C with end-over-end rotation. Beads were then washed three times by centrifugation (300 g, 2 min, 4°C) with lysis buffer and three times with Tris-HCl buffer (i.e., lysis buffer lacking NP-40), dried using a Hamilton syringe, and subjected to analysis by mass spectrometry, as follows.

Beads were washed twice with 25 mM Tris-HCl, pH 8.0, to remove the detergent residuals and subjected to the “on-bead-digestion” protocol. Proteins were denatured and reduced in 8 M Urea, 10 mM DTT, 25 mM Tris-HCl, pH 8.0, and then reduced thiols were alkylated by 55 mM iodoacetamide. The proteins were digested for 16 hrs, acidified, and desalted using C18 Stage tips. The peptides were then loaded onto a 25 cm-long EASY sprayPepMap column (75 μm ID, 2 μm, 100 Å, Thermo Scientific PepMapRSLC) and separated using 60 min gradient of buffer A (0.1% formic acid) and buffer B (80% acetonitrile, 0.1% formic acid) at a flow rate of 0.3 μl/min using nanoflow UHPLC instrument, Ultimate 3000 Dionex (Thermo Fisher Scientific) coupled with Q Exactive Plus mass spectrometer (Thermo Fisher Scientific, Waltham, MA USA). The mass spectrometry measurements were done in the data-dependent mode, as described.^58^ The column was washed with 80% acetonitrile, 0.1% formic acid for 40 min to avoid a carryover of the peptides between the samples. Raw MS files were analyzed with MaxQuant version 1.5.3.12. To identify EspH interacting proteins, the MS/MS spectra were searched against the *Homo Sapiens* and *Escherichia coli* O127 Uniprot FASTA sequences. Only peptides with at least seven amino acids were considered, and the required false discovery rate (FDR) was set to 1% at the peptide and protein level. Protein identification required at least 2 unique or razor peptides per protein. The label-free quantification (LFQ) algorithm was used to quantify differences in protein abundance. Protein contaminations and proteins identified by less than 2 peptides were excluded from the analysis. In addition, only proteins identified in at least two repeats were considered for the analysis. Proteins that were not detected in the control sample or enriched 5-fold with FDR <0.05 were considered EspH binders. We used the STRING server to visualize the protein interaction network (http://string-db.org/), applying stringent parameters for the interaction definitions (highest confidence interaction, experimentally validated, or found in other databases). Graphic representation of the interactions was done using Cytoscape (https://cytoscape.org/).

### Pulldown of endogenous or ectopically expressed HA-tagged ABR with translocated EspH

Pulldown experiments were carried out as described above except that HeLa cells were cultured on 15 cm plates (Thermo Scientific #168381). Cells were infected, lysed, and EspH was pulled down with StAv agarose beads. The precipitated EspH and coprecipitated endogenous ABR were detected by SDS-PAGE followed by IB, using anti-SBP and anti-ABR antibodies, respectively. Similar experiments were performed with HeLa cells transfected with an HA-tagged FL human ABR encoding plasmid for 48 hrs. In these experiments, ABR was detected by IB using anti-HA antibodies.

### Pulldown of HA-tagged ABR with ectopically expressed EspH-eGFP

HeLa cells cultured on 15 cm plates were transfected with eGFP, EspH*_wt_*-eGFP, or EspH_Δ*130-168*_-GFP encoding plasmids. Twenty-four hrs post-transfection cells were washed three times with ice-cold PBS and lysed in ice-cold lysis buffer [10 mM Tris (pH-7.4), 150 mM NaCl, 0.5% NP-40] supplemented with protease and phosphatase inhibitors. Lysates were centrifuged (5,000 g, 15 min, 4°C), and the protein concentration of the supernatants was determined with the BCA protein assay kit. Equal amounts (~5 mg) of cell lysates were incubated with 20 µl of GFP-Trap agarose beads (Chromotek, #gta-10, 50% slurry pre-washed with lysis buffer) for 4 hrs at 4°C with end over end rotation. Beads were then washed 3x with lysis buffer (300 g, 2 min, 4°C), dried using a Hamilton syringe, and analyzed by SDS-PAGE followed by IB.

### Pulldown of endogenous ABR, or recombinantly expressed and purified ABR, with recombinantly expressed and purified EspH-38aa

For analyzing the interactions with endogenous ABR, HeLa cells were cultured on 15 cm plates, washed three times with ice-cold PBS, and then lysed with lysis buffer [50mM Tris (pH-7.4), 150 mM NaCl, 0.5% NP-40] supplemented with protease and phosphatase inhibitors. Lysates were centrifuged (5,000 g, 15 min, 4°C), and the protein concentration of the supernatants was determined with the BCA protein assay kit. Cell lysates (2–3 mg of protein) were exposed to GST-EspH-38aa-6xHis-SBP (200 µg) coupled to StAv beads, pre-washed twice with lysis buffer, for 4 hrs at 4°C with end-to-end rotation. Beads were then washed three times with lysis buffer (300 g, 2 min, 4°C), and precipitated proteins were analyzed by SDS-PAGE followed by IB. To analyze the interactions between recombinant proteins, the EspH-38-6xHis-SBP, 6xHis-TEV-SUMO-ABR-2xFLAG or 6xHis-TEV-SUMO-eGFP were expressed and purified, as described in the Supplementary Methods and Figures S4-5. Purified EspH-38aa-6xHis-SBP (10 µg) was incubated with StAv agarose beads for 4 hrs at 4°C in lysis buffer (50 mM Tris [pH-7.4], 150 mM NaCl, 0.5% NP-40) with end over end rotation. Beads were then washed 3 times in ice-cold lysis buffer. Equal molar concentrations of purified 6xHis-TEV-SUMO-eGFP (40 µg), 6xHis-TEV-SUMO-ABR-2xFLAG (108 µg) proteins were incubated with the pre-coupled EspH-38aa-6xHis-SBP StAv agarose beads for 4 hrs at 4°C. Beads were then washed three times with lysis buffer by centrifugation (300 g, 2 min, 4°C), the precipitated beads were syringe-dried, and proteins were analyzed by SDS-PAGE followed by IB.

### Pulldown of ABR from HeLa cell lysates by GST-EspH38aa-6xHis-SBP coupled to glutathione beads

GST-EspH-38aa-6xHis-SBP in the pooled E1-4 fractions (Figure S4a) were incubated with StAv beads for 4 hrs at 4°C, washed and stored in storage buffer as described above. HeLa cells were cultured on 15 cm plates were lysed as above. Cell lysates (2–3 mg of protein) were exposed to GST-EspH-38aa-6xHis-SBP coupled StA Agarose beads (200 µg of the protein), pre-washed twice with lysis buffer, for 4 hrs at 4°C with end to end rotation. Beads were then washed three times with lysis buffer by centrifugation (3500 rpm, 2 min, 4°C), and proteins precipitated with the beads were detected by IB, using anti-SBP (EspH) and anti-ABR antibodies, respectively.

### Fluorescence microscopy

Immunofluorescence labeling of cells was performed as described.^58^ Briefly, cells were fixed with 4% formaldehyde at 22°C for 20 min. Subsequently, cells were washed 3 times with 1xPBS and stained with indicated primary and secondary antibodies for 1 hr at 37°C. Typically, cells were also stained with Phalloidin [Texas Red (Invitrogen T7471) and CF 647 (Biotium 00041); to visualize filamentous actin (F-actin)] and 4’,6-diamino-2-phenylindole (DAPI, Sigma D9542; to visualize bacteria and cell’s DNA). Cells were mounted and visualized using an Olympus FV-1200 laser scanning confocal microscope equipped with a 60× oil immersion objective (numerical aperture, 1.42). Confocal sections were acquired at *z*-axis intervals of 0.5 μm. The images were analyzed in Fiji (NIH).^58^ A maximal-intensity projection was generated for each stack. For colocalization analyses, 20–30-line intensity profiles were generated, and colocalization analysis was performed, as described.^25,59^ Notably, images taken for colocalization analysis were acquired under identical conditions. Data are presented as percent of colocalized fluorescence peaks derived from approximately 20 intensity profiles.

### Rac1 and Cdc42 GTPase activity assay

The activity levels of Rac1/Cdc42 were estimated by pulldown assay using the p21 binding domain (PBD) of a human p21 activated kinase 1 (PAK1) protein fused to GST [pGEXTK-Pak1 70–117 (Addgene # 12217)] coupled to Glutathione-Agarose (Sigma G4510) beads (GST-PBD beads), as described.^59,60^

### Bacterial invasion assay

The assay was essentially performed as described.^61^ HeLa cells (40,000 cells/ml) were cultured in a 12-well plate for 48 hrs. Cells were washed 2x with PBS and infected with pre-activated bacteria (1 ml/well) for 90 min at 37°C. Immediately after that, the medium bathing the cells was replaced with fresh DMEM supplemented with gentamycin (100 µg/ml; Sigma Aldrich, #G1264) and incubated for 90 min at 37°C in a CO_2_ incubator. Cells were then washed 3x with PBS, the buffer was aspirated, and 1 ml of ice-cold lysis buffer [10 mM Tris-HCl pH 7.4; 1% v/v TritonX-100] was added to each well. Cells were lysed by a few rounds of up and down pipetting, and lysates were subjected to 2-fold serial dilutions (from 1:500 to 1:8000). A fraction of 100 µl from each dilution was plated on LB-agar plates. After 24 hrs, bacterial colonies were counted and multiplied by the corresponding dilution to determine colony-forming units (CFU/ml). The protein concentration of lysates, determined with the BCA-reagent kit (Thermo Scientific, #23227), was ~150 µg/ml for all samples. Plates with 10–300 colonies were counted. Control cells (not treated with gentamycin) showed in all cases a similar colony number (~8x10^6^ CFU/ml), were used for estimating the total cell-associated bacteria.

### Filopodium formation

HeLa cells (0.05X10^6^ cells/well) were seeded on coverslips placed in a 24 well plate and incubated for 24 hrs in a CO_2_ incubator. Cells were then washed 2x with plain DMEM and infected with pre-activated bacteria (0.5 ml/well) for 15 min at 37°C. Thereafter, cells were washed 3x with PBS, fixed, permeabilized, stained with Texas Red Phalloidin (F-actin), DAPI (DNA), immunostained with anti-SBP antibodies followed by anti-mouse AlexaFluor 488 secondary antibodies (EspH), and processed for confocal microscopy, as above.

### SDS-PAGE and immunoblotting (IB)

SDS-PAGE and IB were performed as described.^59^

### Statistical analysis

The GraphPad Prism v. 8.4.3 software was used for statistical analysis.

## Supplementary Material

Supplemental Material

## Data Availability

The authors confirm that the data supporting the findings of this study are available within the article
