# Supplementary material for: EspH interacts with the host active Bcr related (ABR) protein to suppress RhoGTPases
Source: Gut Microbes. 2022 Oct 11;14(1):2130657. doi: 10.1080/19490976.2022.2130657 (PMC9559323; doi:10.1080/19490976.2022.2130657)
Supplement: Supplemental Material [file KGMI_A_2130657_SM3515.zip › Supplementary methods and figs R3 (1).pptx]

## Slide 1
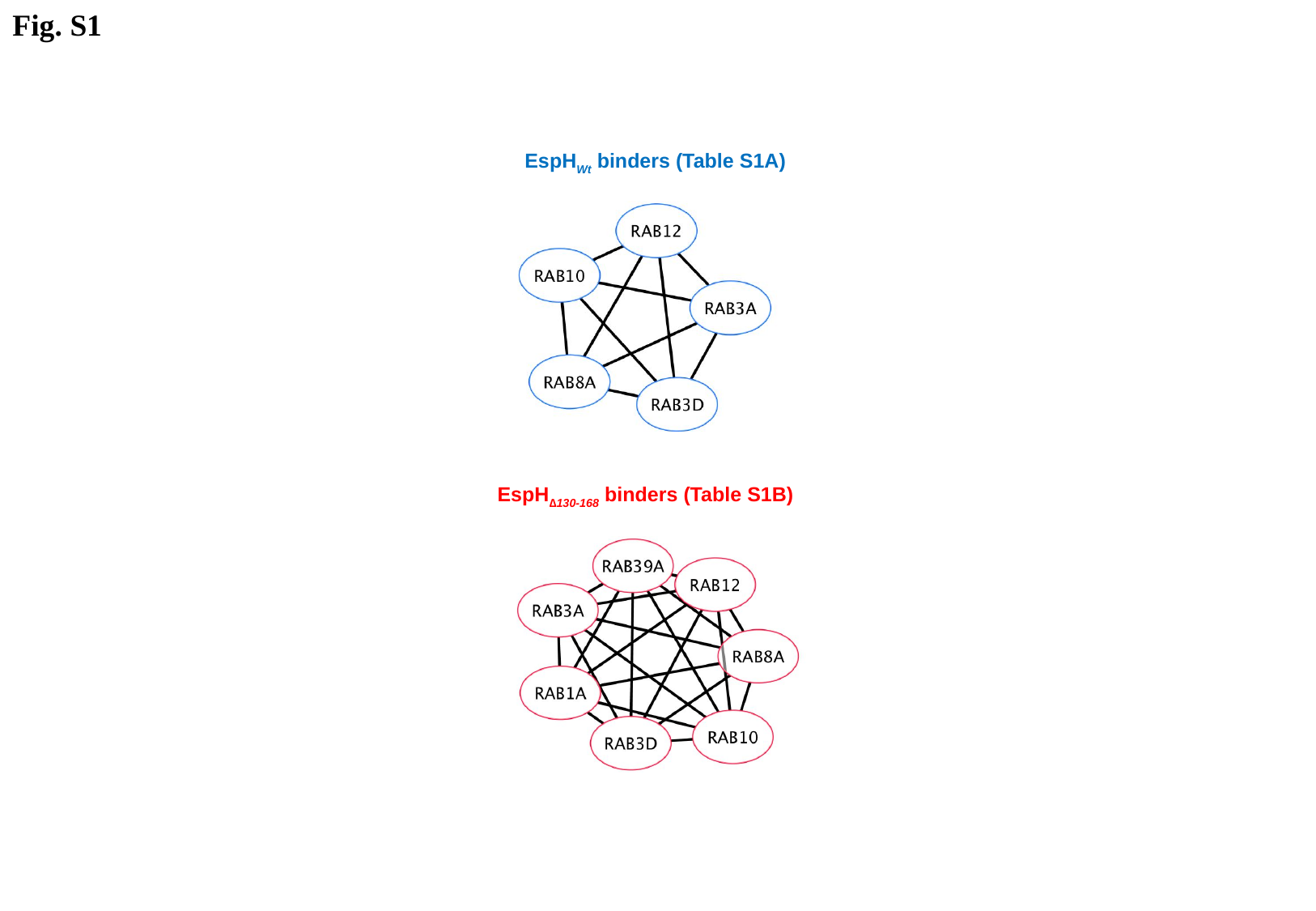

Fig. S1
EspHWt binders (Table S1A)
EspH∆130-168 binders (Table S1B)

## Slide 2
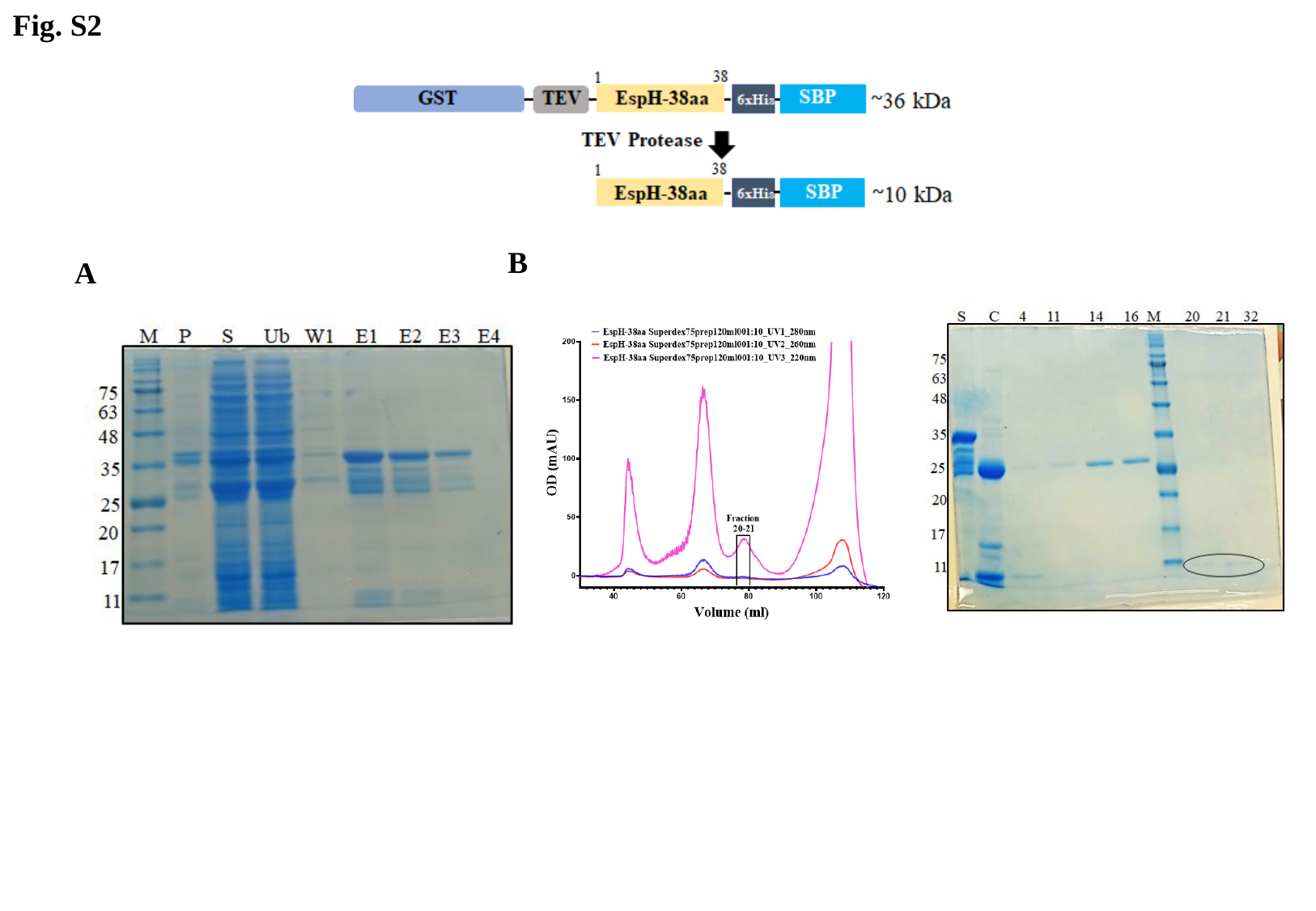

Fig. S2
B
A

## Slide 3
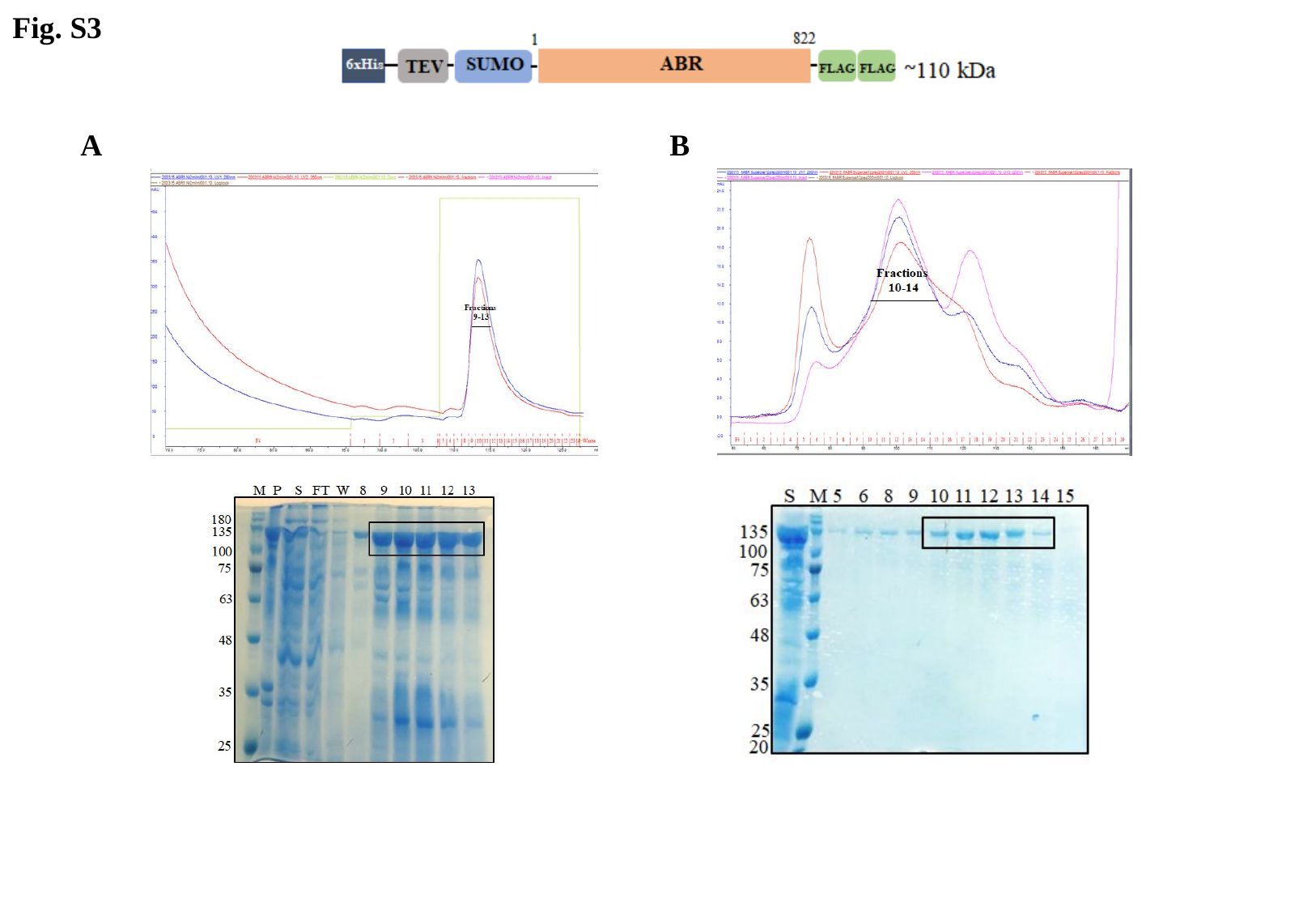

Fig. S3
A
B

## Slide 4
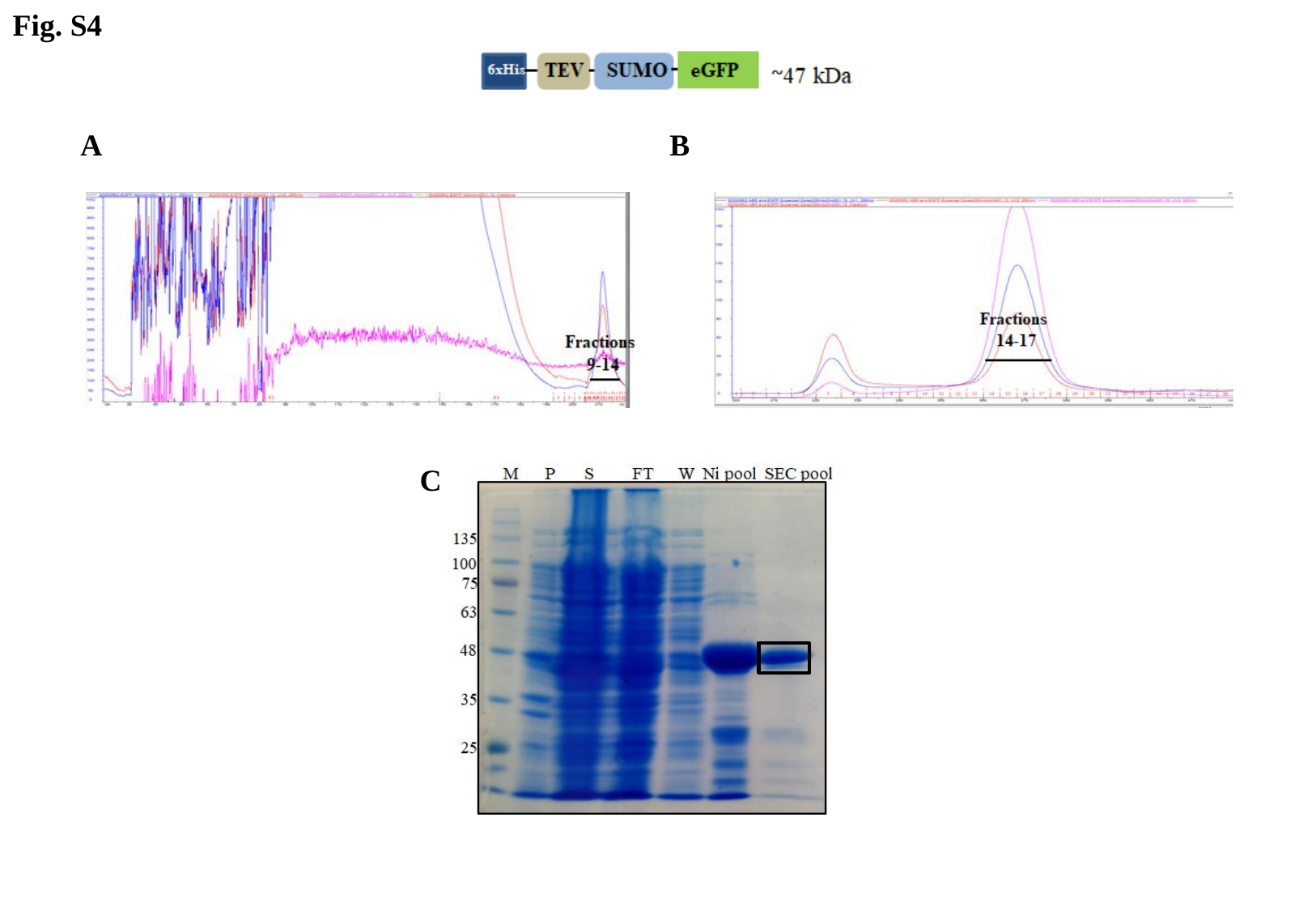

Fig. S4
A
B
C

## Slide 5
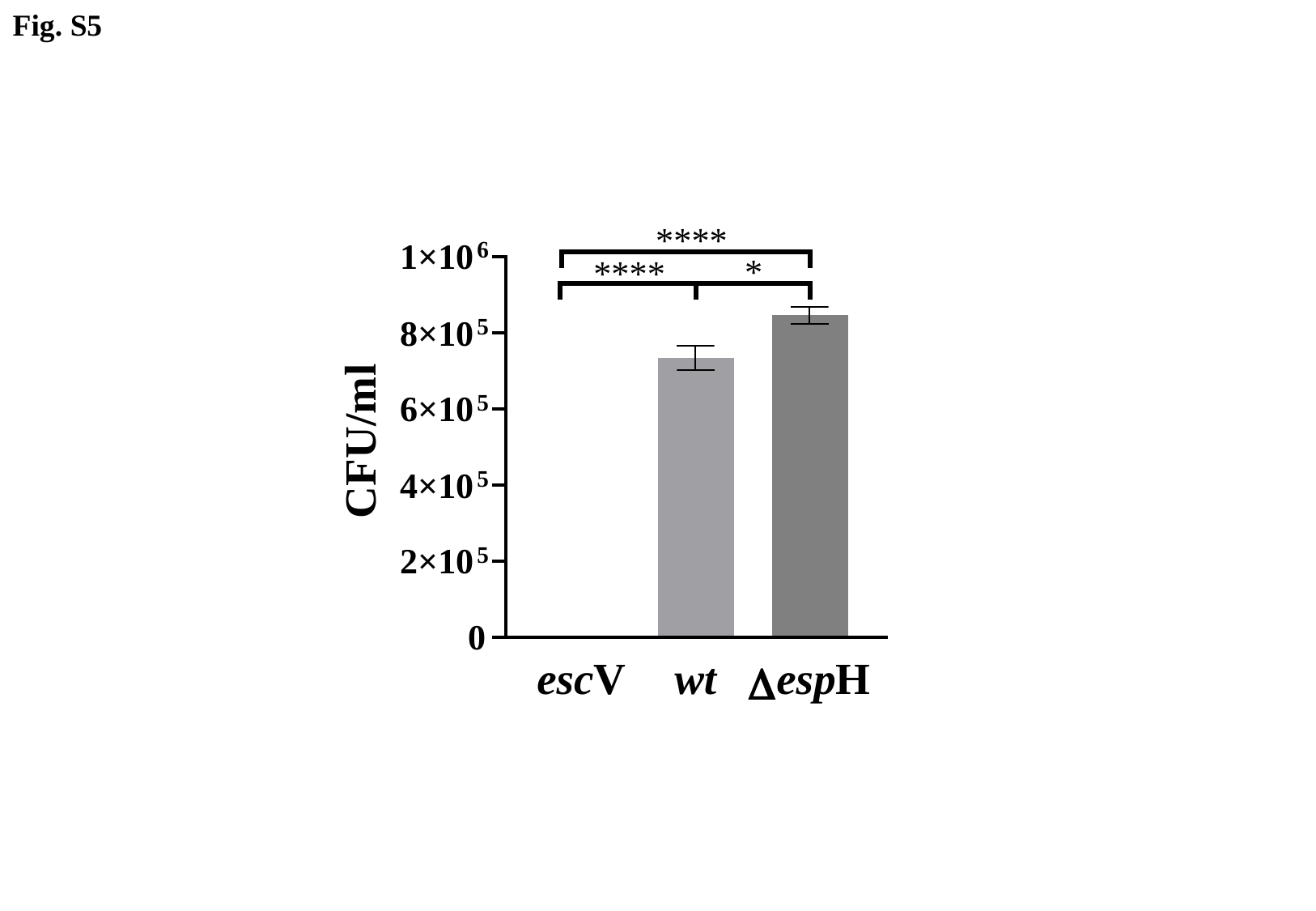

Fig. S5

## Slide 6
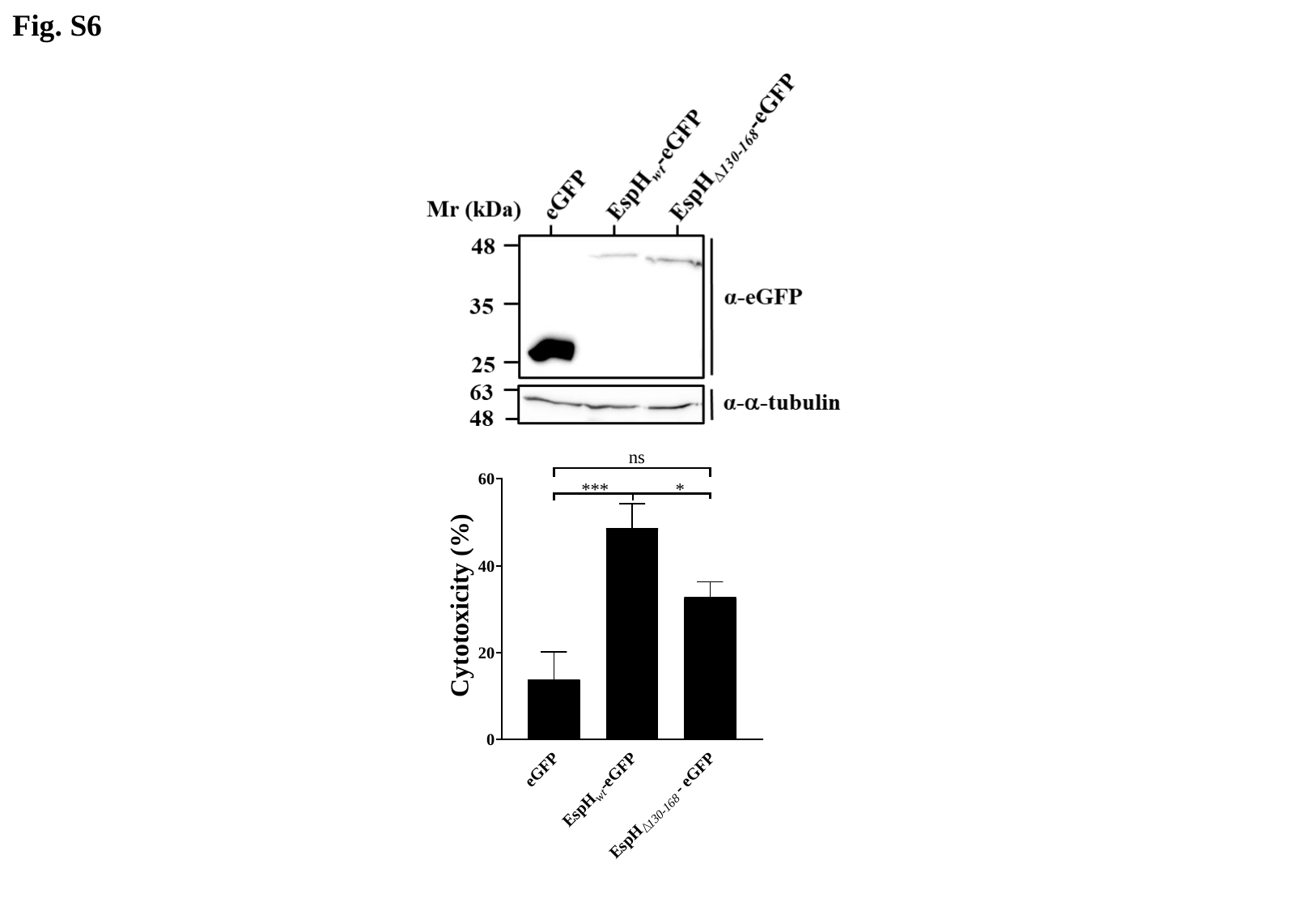

Fig. S6

## Slide 7
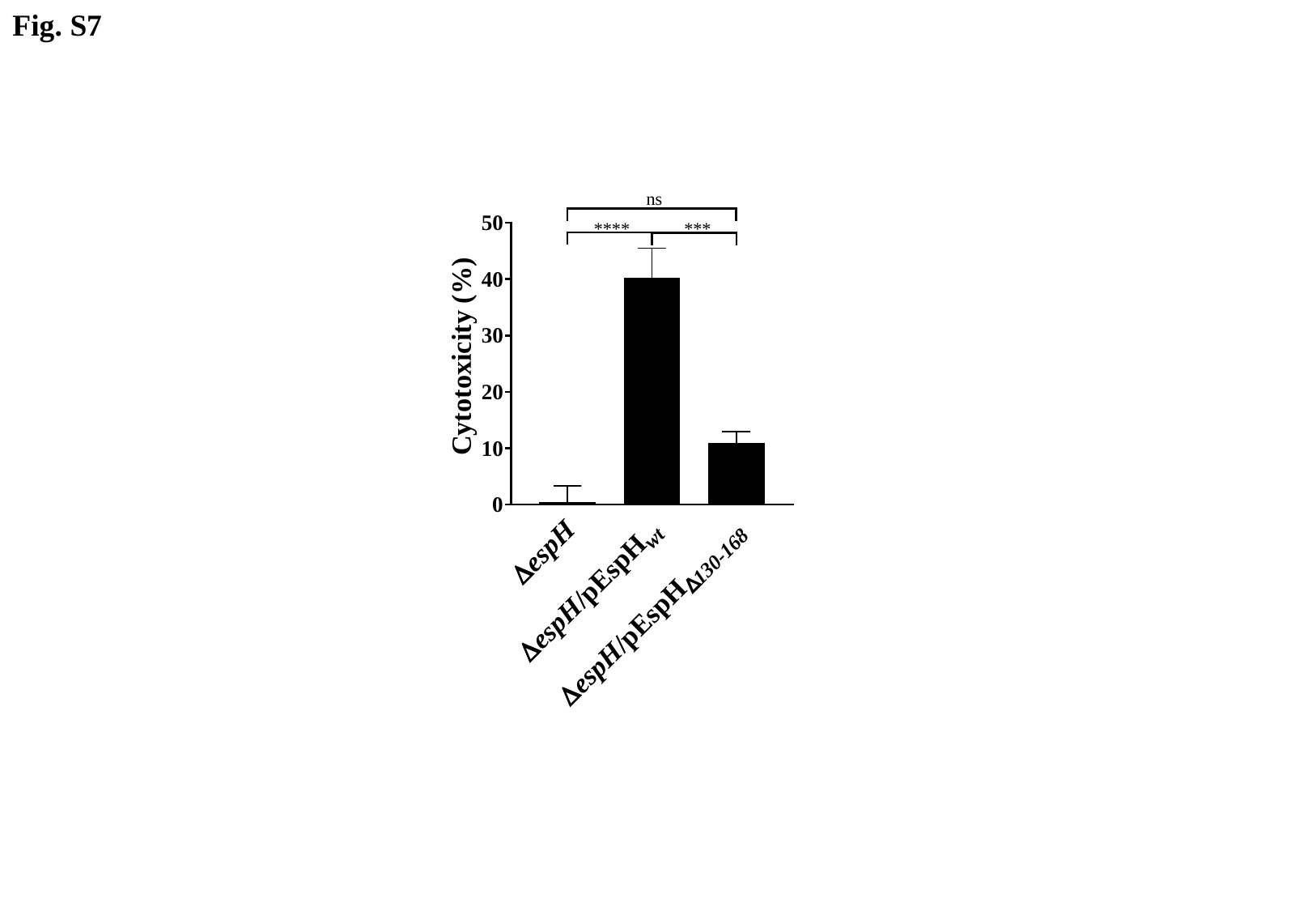

Fig. S7
