# Supplementary material for: EspH interacts with the host active Bcr related (ABR) protein to suppress RhoGTPases
Source: Gut Microbes. 2022 Oct 11;14(1):2130657. doi: 10.1080/19490976.2022.2130657 (PMC9559323; doi:10.1080/19490976.2022.2130657)
Supplement: Supplemental Material [file KGMI_A_2130657_SM3515.zip › Supplementary_Tables_R3_C.docx]

**Supplementary Tables**

**Table S1A: Human proteins identified to be enriched in pulldown experiments of cells infected with EPEC-Δ*espH*/pEspH*_wt_* vs. EPEC-Δ*espH***

| Uniprot ID | Gene name | Protein name | Fold change relative to *espH* |
| --- | --- | --- | --- |
| **Q12979** | **ABR** | **Active breakpoint cluster region-related protein** | **>1000** |
| Q6IQ22 | RAB12 | Ras-related protein Rab-12 | 100.48 |
| P20336 | RAB3A | Ras-related protein Rab-3A | 75.38 |
| O95716 | RAB3D | Ras-related protein Rab-3D | 60.55 |
| P61006 | RAB8 | Ras-related protein Rab-8A (Oncogene c-mel) | 41.96 |
| Q96AB3 | ISOC2 | Isochorismatase domain-containing protein 2 | 18.63 |
| P61026 | RAB10 | Ras-related protein Rab-10 | 17.25 |
| Q7Z7L7 | ZER1 | Protein zer-1 homolog (Hzyg) (Zyg-11 homolog B-like protein) (Zyg11b-like protein) | 14.54 |
| Q9NVI1 | FANCI | Fanconi anemia group I protein | 6.89 |
| P22234 | ADE2 | Multifunctional protein ADE2 [Includes: Phosphoribosylaminoimidazole-succinocarboxamide synthase (EC 6.3.2.6) (SAICAR synthetase); Phosphoribosylaminoimidazole carboxylase (EC 4.1.1.21) (AIR carboxylase) (AIRC)] | 6.45 |
| O14980 | XPO1 | Exportin-1 (Exp1) (Chromosome region maintenance 1 protein homolog) | 5.65 |
| Q13501 | SQSTM1 | Sequestosome-1 (EBI3-associated protein of 60 kDa) (EBIAP) (p60) (Phosphotyrosine-independent ligand for the Lck SH2 domain of 62 kDa) (Ubiquitin-binding protein p62) | 5.18 |

**Table S1B: Human proteins identified to be enriched in pulldown experiments of cells infected with EPEC-Δ*espH*/pEspH_∆_*_130-168_* vs. EPEC-Δ*espH* (i.e. EspH_∆_*_130-168_* interacting proteins)**

| Uniprot ID | Gene name | Protein name | Fold change relative to *espH* |
| --- | --- | --- | --- |
| P61026 | RAB10 | Ras-related protein Rab-10 | 427.79 |
| P61006 | RAB8A | Ras-related protein Rab-8A (Oncogene c-mel) | 126.82 |
| Q14964 | RAB39A | Ras-related protein Rab-39A (Rab-39) | 118.78 |
| O95716 | RAB3D | Ras-related protein Rab-3D | 81.73 |
| P62820 | RAB1A | Ras-related protein Rab-1A (YPT1-related protein) | 78.38 |
| Q6IQ22 | RAB12 | Ras-related protein Rab-12 | 45.02 |
| Q9H0U4 | RAB1B | Ras-related protein Rab-1B | 25.64 |
| P20336 | RAB3A | Ras-related protein Rab-3A | 24.18 |
| P16070 | CD44 | CD44 antigen / Extracellular matrix receptor III | 10.53 |
| Q7Z7L7 | ZER1 | Protein zer-1 homolog (Hzyg) (Zyg-11 homolog B-like protein) (Zyg11b-like protein) | 7.84 |
| P55060 | XPO2 | Exportin-2 (Exp2) (Cellular apoptosis susceptibility protein/Chromosome segregation 1-like protein | 7.19 |

**Table S1C: Differentially abundant proteins pulled down with EspH*_wt_* vs. EspH_∆_*_130-168_***

| Uniprot ID | Gene name | Protein name | Fold change relative to EspH_∆_*_130-168_* |
| --- | --- | --- | --- |
| **Increased in the WT pulldown experiment relative to EspH_∆_*_130-168_*** | | | |
| **Q12979** | **ABR** | **Active breakpoint cluster region-related protein** | **62.35** |
| P62072 | TIM10 | Mitochondrial import inner membrane translocase subunit Tim10 | 8.10 |
| Q01844 | EWSR1 | RNA-binding protein EWS (EWS oncogene) (Ewing sarcoma breakpoint region 1 protein) | 6.71 |
| Q7Z7L7 | ZER1 | Protein zer-1 homolog (Hzyg) (Zyg-11 homolog B-like protein) (Zyg11b-like protein) | 3.65 |
| P11021 | GRP78 | Endoplasmic reticulum chaperone BiP (78 kDa glucose-regulated protein) | 2.20 |
| **Decreased in the WT pulldown experiment vs. EspH_∆_*_130-168_*** | | | |
| Q9H0U4 | RAB1B | Ras-related protein Rab-1B | 23.25 |
| P16070 | CD44 | CD44 antigen / Extracellular matrix receptor III | 12.92 |
| P62820 | RAB1A | Ras-related protein Rab-1A (YPT1-related protein) | 12.15 |
| O76031 | CLPX | Mitochondrial ATP-binding subunit clpX-like | 6.33 |
| P62249 | RPS16 | 40S ribosomal protein S16 | 4.61 |
| P61026 | RAB10 | Ras-related protein Rab-10 | 4.39 |
| P46783 | RPS10 | 40S ribosomal protein S10 | 4.09 |
| Q6IQ22 | RAB12 | Ras-related protein Rab-12 | 4.01 |
| Q8JGS9 | RPS18 | 40S ribosomal protein S18 | 3.85 |
| P49411 | TUFM | Elongation factor Tu, mitochondrial (EF-Tu / P43) | 3.27 |
| Q96RP9 | GFM1/EF1 | Mitochondrial Elongation factor G 1 | 2.42 |
| P61006 | RAB8A | Ras-related protein Rab-8A (Oncogene c-mel) | 2.42 |
| P17987 | TCP1 | T-complex protein 1 subunit alpha (TCP-1-alpha) | 1.95 |

**Table S2: Bacterial EPEC strains (all mutant strains are derivatives of E2348/69)**

| **Strain name (number)** | **Description** | **Source/Reference** | **Comments** |
| --- | --- | --- | --- |
| Δ*espH* (XT111) | *espH::Kan^r^* | ^1, 2^ | EPEC bearing mutationally inactivated in the LEE gene *espH* |
| Δ*espH/*pEspH*_wt_* (BA1104) | ∆*espH* strain complemented with pSA10-EspH*_wt_* encoding plasmid | ^1^ | EspH tagged with 6XHis-SBP at the C-terminus |
| Δ*espH/*pEspH*_∆130-168_* (BA1139) | ∆*espH* strain complemented with pSA10-EspH*_∆130-168_* encoding plasmid | ^1^ | EspH whose C-terminal 38aa segment has been deleted and tagged with 6XHis-SBP |
| E2348/69 (*wt*)  BA140 | EPEC-*wt* isolate, serotype O127:H6, NA^r^ | ^3^ | EPEC-*wt* isolate |
| SN191(*escV*) BA253 | *escV*::*miniTn5Kan^r^* | ^4^ | EPEC mutated in the T3SS |

**Table S3: Primary and secondary antibodies**

| **Antibody** | **Primary/Secondary**  **dilution (IF/WB)** | **Description** |
| --- | --- | --- |
| Rabbit anti-ABR | Primary  1:1000 (WB) | Monoclonal anti- human ABR antibody [EPR7657]; ab167420; Abcam |
| Mouse anti-SBP tag | Primary  1:250 (IF)  1:2000 (WB) | Streptavidin binding peptide (SBP) antibody (SB19-C4):sc101595; Santa Cruz Biotechnology |
| Rabbit anti-FLAG antibody | Primary  1:1000 (WB) | Polyclonal anti-FLAG antibody; F7425; Sigma Aldrich |
| Mouse anti-GADPH | Primary  1:1000 (WB) | Monoclonal anti-GADPH antibody (0411); sc-47724; Santa Cruz Biotechnology |
| Mouse anti-β actin | Primary  1:5000 (WB) | Monoclonal antibody, ACTN05 (C4); ab3280; Abcam |
| Mouse anti-Rac1 | Primary | Monoclonal Purified Anti-Rac1 Clone 102 (RUO); 610650, BD Transduction Laboratories |
| Mouse anti-Cdc42 | Primary  1:250 (WB) | Monoclonal anti-CDC42 Clone44(RUO); 610929, BD Transduction Laboratories |
| Rabbit anti-HA | Primary  1:5000 (WB)  1:200 (IF) | Rabbit polyclonal anti-HA tag antibody - ChIP Grade;Abcam ab9110 |
| Rabbit anti-α-tubulin | Primary  1:2000 (WB) | Mouse monoclonal Anti-α-Tubulin antibody; T6074; Sigma Aldrich |
| Mouse anti-GFP | Primary  1:350 (WB) | Mouse monoclonal Anti-GFP (B-2) antibody; sc-9996; Santa Cruz Biotechnology |
| Donkey anti-mouse Cy5 (Alexa Fluor 647) | Secondary  1:300 (IF) | Cy5 - Donkey Anti-Mouse IgG; Jackson ImmunoResearch Laboratories |
| Goat anti-mouse IgG, Alexa Fluor 488 | Secondary  1:300 (IF) | Alexa Fluor 488- AffiniPure Goat Anti-Mouse IgG; 115-545-062; Jackson ImmunoResearch Laboratories |
| Goat anti-rabbit, IgG, Alexa Fluor 594 | Secondary  1:300 (IF) | Alexa Fluor 594 - Goat Anti-Rabbit IgG; A11037; Molecular Probes; Invitrogen; |
| Goat anti-Rabbit IgG, Alexa Fluor 488 | Secondary  1:300 (IF) | Alexa Fluor 488 - Goat Anti-Rabbit IgG; A11008; Life technologies |
| Peroxidase goat anti-mouse IgG | Secondary  1:10000 (WB) | Peroxidase- AffiniPure Goat Anti-Mouse IgG; 115-035-166; Jackson ImmunoResearch Laboratories |
| Peroxidase goat anti-rabbit IgG | Secondary  1:10000 (WB) | Peroxidase- AffiniPure Goat Anti-Rabbit IgG; 111-035-003; Jackson ImmunoResearch Laboratories |

**Table S4: Plasmids**

| **Plasmid name** | **Description** | **Reference** |
| --- | --- | --- |
| pGST parallel 1 vector  (BA805) | Bacterial expression IPTG inducible vector, Amp^r^ | A kind gift from Dr. Sheffield, PJ, and Prof. Derewenda ZS, University of Virginia (UVA) |
| pGST-EspH38aa-6xHis-SBP  (BA756) | pGST 1 vector encoding C-terminal 38aa of EspH comprising 6x His and an SBP tag in the C-terminus (**Fig. S3**) | This study |
| 6xHis-SUMO-ABR-2xFlag  (BA757) | SUMO vector encoding full-length ABR protein comprising 2x Flag tag in the C-terminus (**Fig. S4**) | This study |
| pETM11 Sumo3 eGFP  (BA797) | Bacterial expression IPTG inducible vector, Kan^r^ (**Fig. S5**) | A kind gift from Dr. Huseyin Besir, Protein Expression and Purification Core Facility, EMBL |
| FL-ABR  (BA729) | Mammalian expression pKH3 vector encoding full-length human ABR protein isoform B [NP_001083.2 (822 aa)], bearing 3xHA tag in its N-terminus | Addgene plasmid, #38190 |
| DH-PH  (BA801) | Mammalian expression pKH3 vector encoding the DH-PH domain of ABR protein, with 3xHA tag in the N-terminus | This study |
| C2  (BA802) | Mammalian expression pKH3 vector encoding the C2 domain of ABR protein deleted, with 3xHA tag in the N-terminus | This study |
| GAP*_wt_*  (BA803) | Mammalian expression pKH3 vector encoding the GAP domain of ABR protein, with 3xHA tag in the N-terminus | This study |
| ∆DH-PH  (BA798) | Mammalian expression pKH3 vector encoding ABR protein deleted of DH-PH domain, with 3xHA tag in the N-terminus | This study |
| ∆C2  (BA799) | Mammalian expression pKH3 vector encoding ABR protein deleted of C2 domain, with 3xHA tag in the N-terminus | This study |
| ∆GAP  (BA800) | Mammalian expression pKH3 vector encoding ABR protein deleted of GAP domain, with 3xHA tag in the N-terminus | This study |
| EspH-eGFP  (pAA6271) | Mammalian expression pEGFP-N1 vector encoding EspH, with eGFP tag in the C-terminus | ^1^ |
| EspH*_∆130-168_*-eGFP  (pAA6290) | Mammalian expression pEGFP-N1 vector encoding EspH 1-129aa, with eGFP tag in the C-terminus | ^1^ |
| peGFP-N1  (BA450) | eGFP vector | CloneTech Laboratories #6085-1 |
| pGEXTK-Pak1 70-117 (GST-PBD) | A bacterial expression vector encoding the p21 binding domain (PBD) of a human p21 activated kinase 1 (PAK1) protein fused to GST | Addgene # 12217 |
| GAP*_R646A/N758A_*  (BA853) | Mammalian expression pKH3 vector encoding the GAP domain with double mutation R683A and N795A of ABR protein, with 3xHA tag in the N-terminus | This study |
| pLKO.1-Puro  (BA895) | Mammalian Lentiviral cloning vector pLKO.1-Puro vector used for cloning ABR shRNA | Addgene # 8453 |
| pLKO.1-Puro-ABR shRNA.1.2  (BA887) | Lentiviral vector pLKO.1-Puro vector encoding the ABR shRNA used for silencing ABR expression | This study |
| pLKO.1-Puro-Scramble  (BA391) | Lentiviral vector pLKO.1-Puro vector encoding the Scramble RNA sequence used as a negative control | Addgene # 1864 |
| psPAX2  (BA893) | Packaging plasmid for producing viral particles | Addgene # 12260 |
| pMD2.G  (BA894) | Envelope plasmid for producing viral particles | Addgene # 12259 |

**Table S5. List of primers and their usage**

| **S. No.** | **Name** | **Sequence** | **Usage** |
| --- | --- | --- | --- |
| 1F' | F pGST1 linear | taaGGAATTCAAAGGCCTACGTCGACGAG | Generation of GST-EspH-6xHis-SBP |
| 2R' | R pGST1 linear | tGGATCCATGGCGCCCTGAAAATAC | Generation of GST-EspH-6xHis-SBP |
| 3F' | F GA 38AA-GST | TTCAGGGCGCCATGGATCCaGAAGGCTTTTTAACTGAAAGAGGGCTCTC | Generation of GST-EspH-6xHis-SBP |
| 4R' | R GA 38AA-GST | GTAGGCCTTTGAATTCCttaCGGTTCACGCTGACCCTGCG | Generation of GST-EspH-6xHis-SBP |
| 5 | 3’pGEX | CCG-GGAGCTGCATGTGTCAGAGG | Verification of GST-EspH-6xHis-SBP |
| 6 | pEEF3 | GGG CGA CAC GGA AAT GTT G | Verification of GST-EspH-6xHis-SBP |
| 7F' | F Linear sumo | AAGCTTGCGGCCGCACTCGAG | Generation of His-SUMO -ABR-2xFlag |
| 8R' | R Linear sumo | TCCACCGGTCTGTTGCTGGA | Generation of His-SUMO -ABR-2xFlag |
| 9F' | F GA ABR | TCCAGCAACAGACCGGTGGAATGGAGGAGGAAGAGGAGGCG | Generation of His-SUMO -ABR-2xFlag |
| 10R' | R GA ABR | CTTGAGTTCTGCGAAGGAAATGGGGGGGTGCTGCAGGTAGTAG | Genartion of His-SUMO -ABR-2xFlag |
| 11 | Gene fragment of Flag tag | TTTCCTTCGCAGAACTCAAGCGGAACACACTGTACTTCTCCACCGACGTGGATTACAAAGATGACGATGATAAGGCTGATTACAAGGATGACGATGACAAGTGAAAGCTTGCGGCCGCACTCGAG | Generation of His-SUMO -ABR-2xFlag |
| 12 | T7 | TAATACGACTCACTATAGGG | Verification of His-SUMO -ABR-2xFlag |
| 13 | T7 Term | GCTAGTTATTGCTCAGCGG | Verification of His-SUMO -ABR-2xFlag |
| 14F' | ABR F | AACTTCCTGTCCAGCATCAAC | Verification of His-SUMO -ABR-2xFlag |
| 15R' | ABR R | CACCTCCTCCACACACTGCCGGA | Verification of His-SUMO -ABR-2xFlag |
| 16F' | DH-PH delta F | AAGGATCTCCAGGCCTTTGTCCTGAG | Generation of HA-ABR_∆DH-PH_ |
| 17R' | DH-PH delta R | CTCCAGGCCTTTCCCTGCTTC | Generation of HA-ABR_∆DH-PH_ and HA-ABR_GAP_ |
| 18F' | C2 delta F | ATCCAGCTGGACCCACAAAC | Generation of HA-ABR_∆C2_ and HA-ABR_GAP_ |
| 19R' | C2 delta R | GAGATCCTTCTTCTGTAGTTTCTGA | Generation of HA-ABR_∆C2_ and HA-ABR_DH-PH_ |
| 20F' | GAP delta F | GCAGAACTCAAGCGGAACAC | Generation of HA-ABR_∆GAP_ and HA-ABR_DH-PH_ |
| 21R' | GAP delta R | CACGGTTTGTGGGTCCAGCTG | Generation of HA-ABR_∆GAP_ |
| 22F' | Linear C2 F | CATGGGCAAAGGACAGGCAGAACTCAAGCGGAACACAC | Generation of HA-ABR_C2_ |
| 23R' | Linear C2 R | CTCAGGACAAAGGCCTGCTCCAGGCCTTTCCCTGCTTCC | Generation of HA-ABR_C2_ |
| 24F' | C2 domain GA F | CAGGCCTTTGTCCTGAG | Generation of HA-ABR_C2_ |
| 25R' | C2 domain GA R | CTGTCCTTTGCCCATGATC | Generation of HA-ABR_C2_ |
| 26F' | N758A F' | TTCACGCCCTGGCTACCGTGTTTG | Generation of HA- GAP*_R646A/N758A_* |
| 26R' | N758A R' | TAGCCAGGGCGTGAAGTGACATTTTG | Generation of HA- GAP*_R646A/N758A_* |
| 27F' | ABR shRNA.1 F' | CCGGGCGTTTGTCGATAACTATAAACTCGAGTTTATAGTTATCGACAAACGCTTTTTG | Generation of pLKO.1-Puro-ABR shRNA.1.2 |
| 28R' | ABR shRNA.2 R' | AATTCAAAAATCCCGTTCAGGATCCACAATCCTCGAGGATTGTGGATCCTGAACGGGA | Generation of pLKO.1-Puro-ABR shRNA.1.2 |
| 29F' | SH_HT_F | GCCGCCCCCTTCACCGAGGGCCTATTTCC | Clone verification of pLKO constructs |
| 30R' | SH_HT_R | CCGGGTGACGCTGCGAACGGACGTGAAGAA | Clone verification of pLKO constructs |

References

1. Ramachandran RP, Vences-Catalan F, Wiseman D, Zlotkin-Rivkin E, Shteyer E, Melamed-Book N, et al. EspH Suppresses Erk by Spatial Segregation from CD81 Tetraspanin Microdomains. Infection and immunity 2018; 86:00303-18.

2. Tu X, Nisan I, Yona C, Hanski E, Rosenshine I. EspH, a new cytoskeleton-modulating effector of enterohaemorrhagic and enteropathogenic Escherichia coli. Mol Microbiol 2003; 47:595-606.

3. Levine MM, Bergquist EJ, Nalin DR, Waterman DH, Hornick RB, Young CR, et al. Escherichia coli strains that cause diarrhoea but do not produce heat-labile or heat-stable enterotoxins and are non-invasive. Lancet 1978; 1:1119-22.

4. Nadler C, Shifrin Y, Nov S, Kobi S, Rosenshine I. Characterization of enteropathogenic Escherichia coli mutants that fail to disrupt host cell spreading and attachment to substratum. Infect Immun 2006; 74:839-49.
