# Supplementary figures and images for: EspH interacts with the host active Bcr related (ABR) protein to suppress RhoGTPases
Source: Gut Microbes. 2022 Oct 11;14(1):2130657. doi: 10.1080/19490976.2022.2130657 (PMC9559323; doi:10.1080/19490976.2022.2130657)

## Slide 1
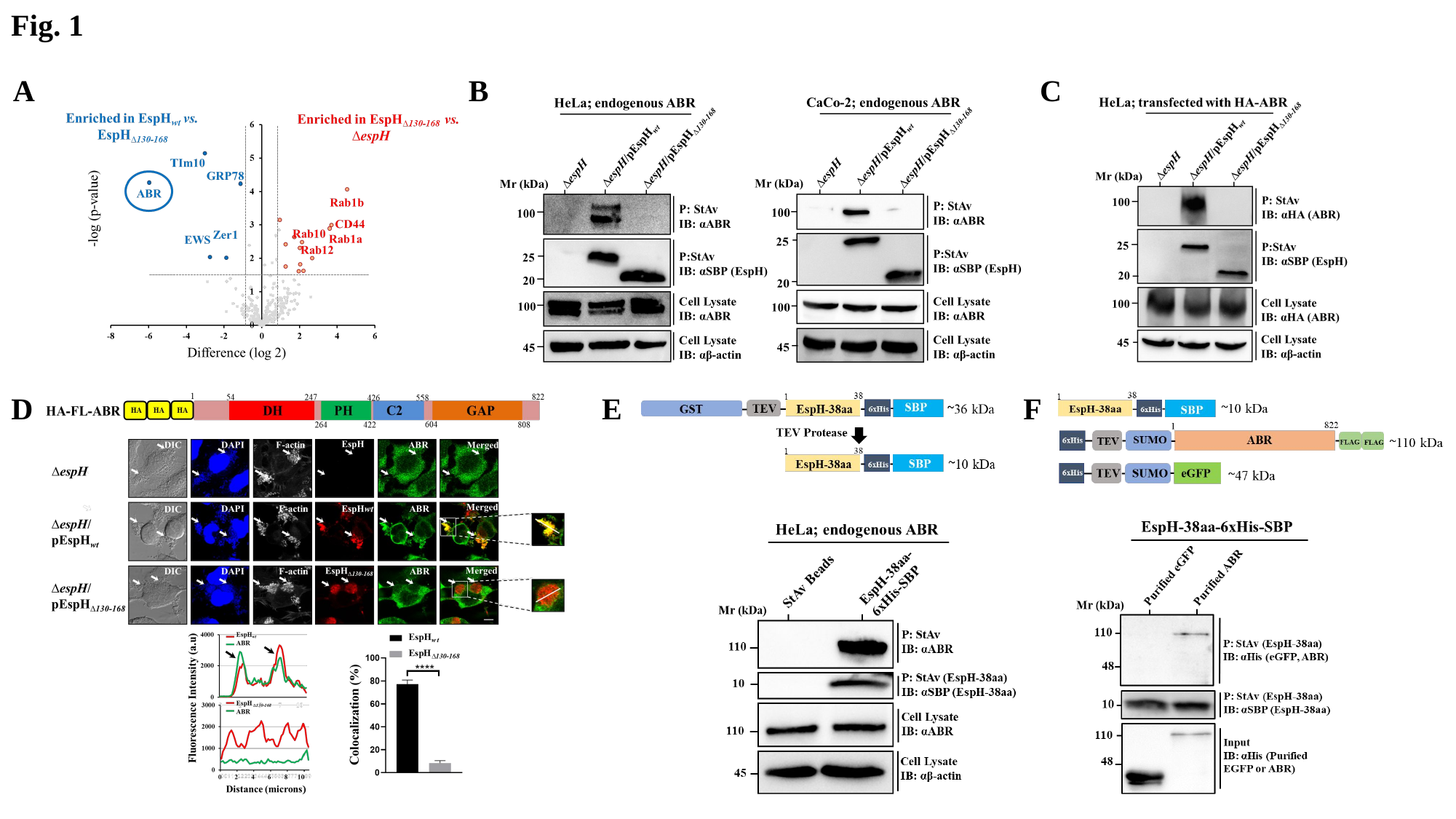

Fig. 1
A
B
C
D
E
F

## Slide 2
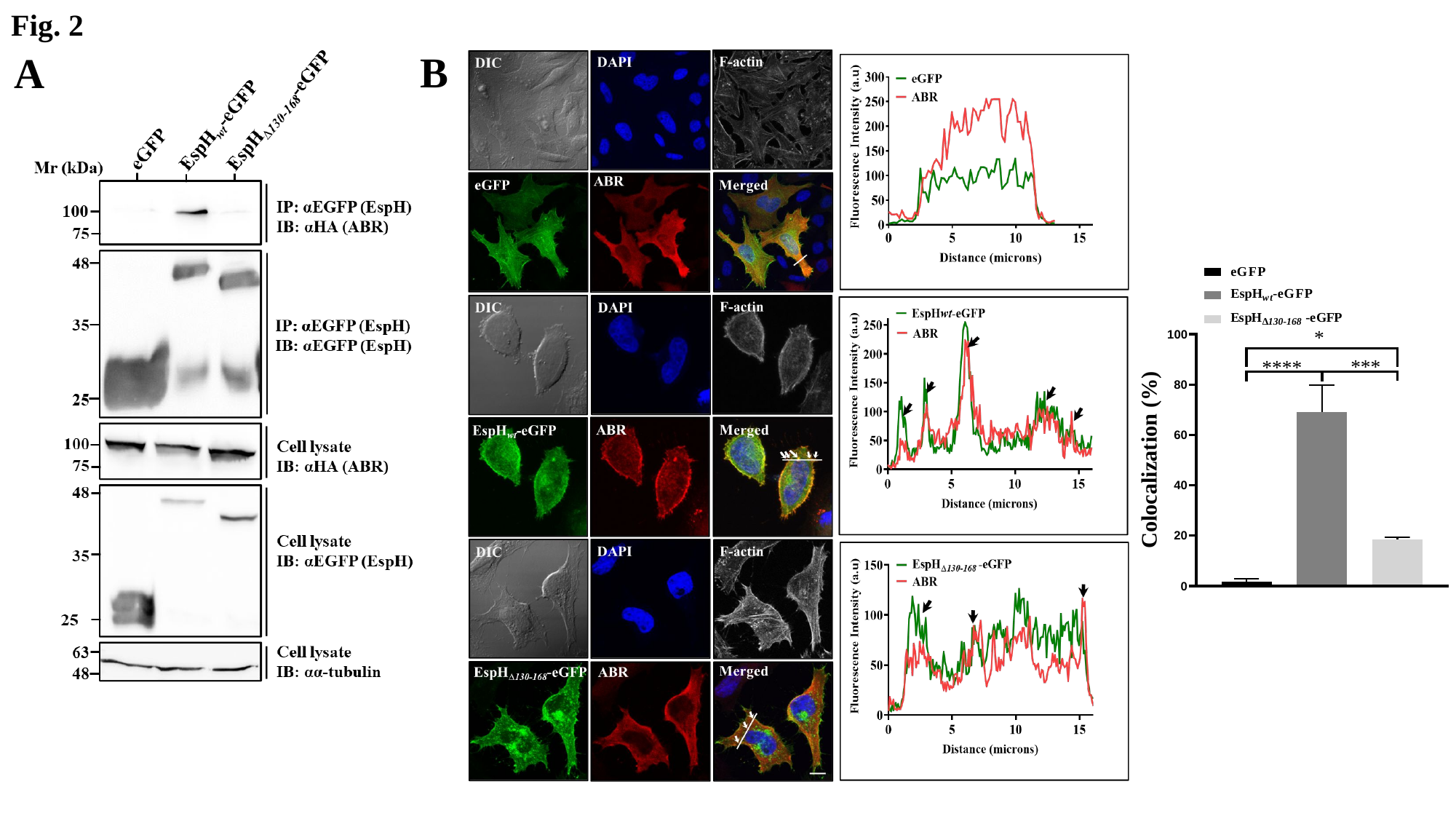

Fig. 2
B
A

## Slide 3
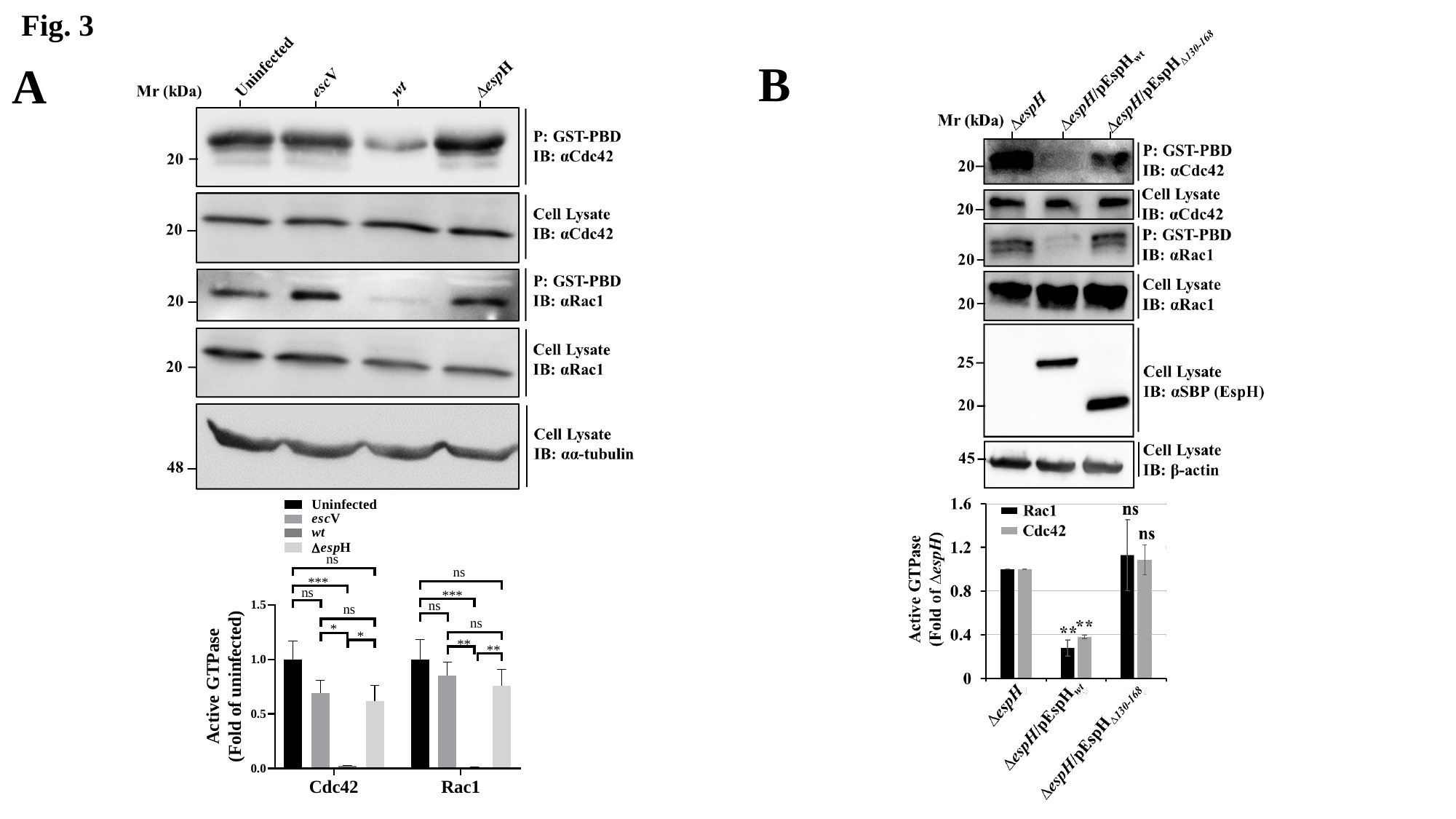

Fig. 3
B
A

## Slide 4
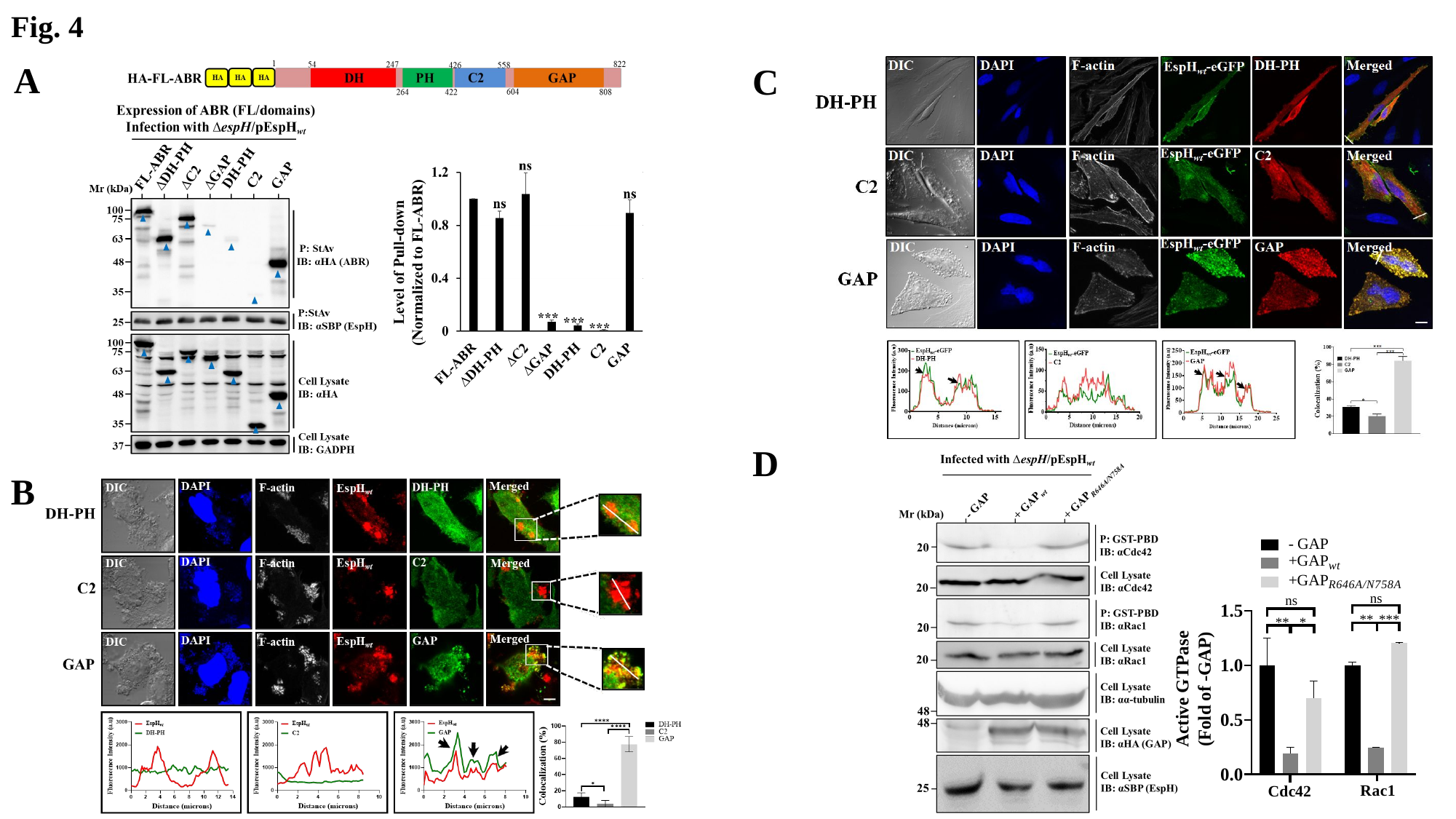

Fig. 4
A
C
D
B

## Slide 5
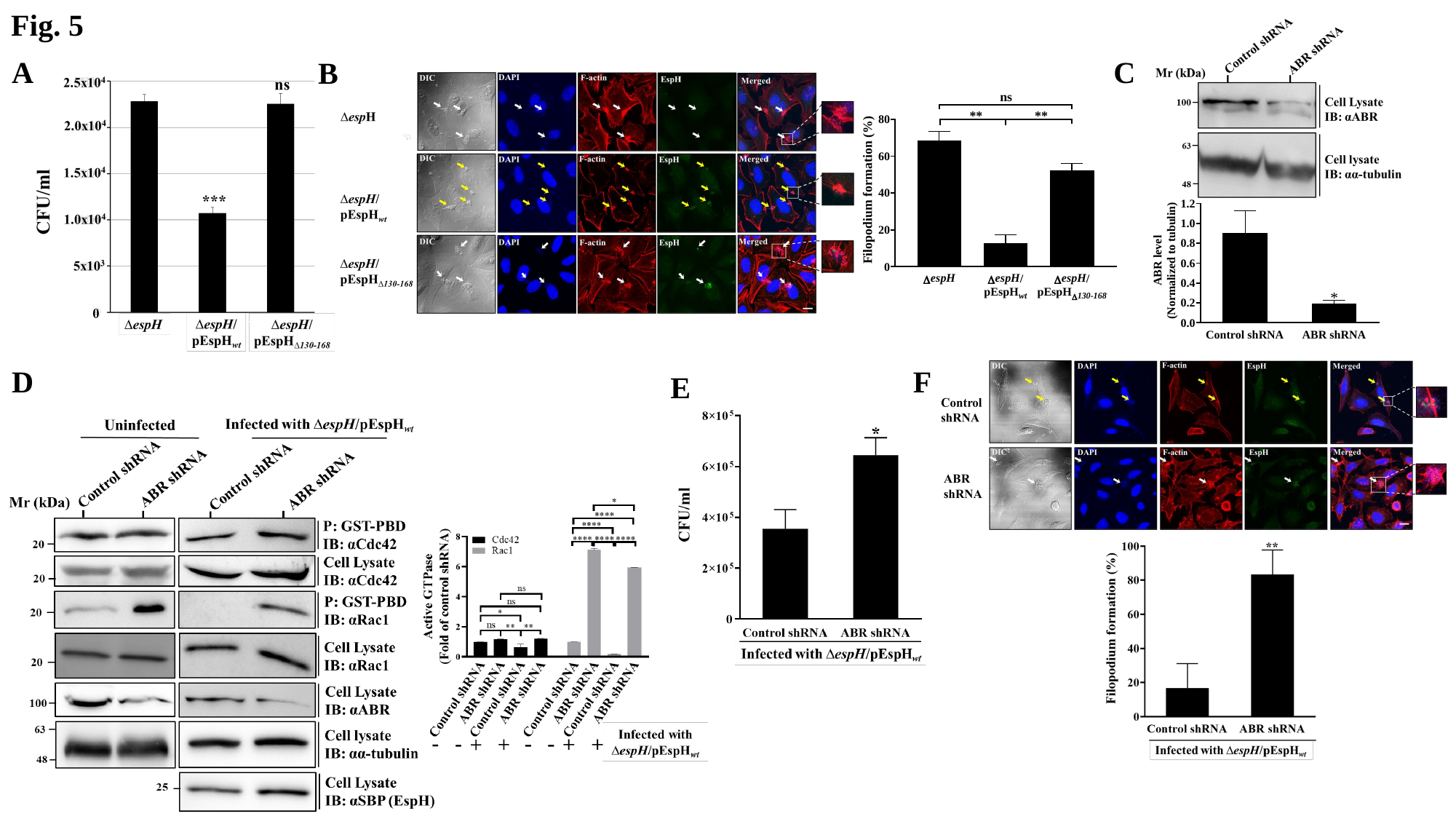

Fig. 5
C
A
B
D
F
E

Supplement: Supplemental Material [file KGMI_A_2130657_SM3515.zip › Ramachandran figs R3 (1).pptx]
